# Systematic and Molecular Basis of the Antibacterial Action of Quinoxaline 1,4-Di-*N*-Oxides against *Escherichia coli*

**DOI:** 10.1371/journal.pone.0136450

**Published:** 2015-08-21

**Authors:** Guyue Cheng, Bei Li, Chenxi Wang, Hongfei Zhang, Guixia Liang, Zhifei Weng, Haihong Hao, Xu Wang, Zhenli Liu, Menghong Dai, Yulian Wang, Zonghui Yuan

**Affiliations:** 1 MOA Laboratory for Risk Assessment of Quality and Safety of Livestock and Poultry Products, Huazhong Agricultural University, Wuhan, Hubei, 430070, China; 2 National Reference Laboratory of Veterinary Drug Residues (HZAU) and MAO Key Laboratory for Detection of Veterinary Drug Residues, Huazhong Agricultural University, Wuhan, Hubei, 430070, China; 3 Hubei Collaborative Innovation Center for Animal Nutrition and Feed Safety, Huazhong Agricultural University, Wuhan, Hubei, 430070, China; Duke University Marine Laboratory, UNITED STATES

## Abstract

Quinoxaline 1,4-di-*N*-oxides (QdNOs) are widely known as potent antibacterial agents, but their antibacterial mechanisms are incompletely understood. In this study, the transcriptomic and proteomic profiles of Esch*erichia coli* exposed to QdNOs were integratively investigated, and the results demonstrated that QdNOs mainly induced an SOS response and oxidative stress. Moreover, genes and proteins involved in the bacterial metabolism, cellular structure maintenance, resistance and virulence were also found to be changed, conferring bacterial survival strategies. Biochemical assays showed that reactive oxygen species were induced in the QdNO-treated bacteria and that free radical scavengers attenuated the antibacterial action of QdNOs and DNA damage, suggesting an oxidative-DNA-damage action of QdNOs. The QdNO radical intermediates, likely carbon-centered and aryl-type radicals, as identified by electron paramagnetic resonance, were the major radicals induced by QdNOs, and xanthine oxidase was one of the QdNO-activating enzymes. This study provides new insights into the action of QdNOs in a systematic manner and increases the current knowledge of bacterial physiology under antibiotic stresses, which may be of great value in the development of new antibiotic-potentiating strategies.

## Introduction

Quinoxaline 1,4-di-*N*-oxide derivatives (QdNOs) have exhibited many diverse biological properties, including antibacterial, antifungal, antiviral, anticancer and insecticidal activities [1]. The QdNOs used in veterinary practice, including quindoxin, carbadox, olaquindox (OLA), mequindox (MEQ) and quinocetone, are known as potent antibacterial agents for the prevention of colibacillosis, diarrhea, and enteric salmonellosis in livestock and poultry [2]. Cyadox (CYA) is a novel QdNO derivative, and the microbiological safety evaluation of CYA suggests that it is a safe member of the QdNO family [3].

Although QdNO antibacterials have been widely used for more than half a century, the mode of action underlying their antibacterial property has not yet been clearly elucidated. Previous investigations have shown that QdNOs can inhibit DNA synthesis and cause DNA damage [4, 5]. Suter *et al*. [4] has suggested that some undefined bacterial reductase(s) are involved in the metabolic activation of QdNOs and that some unstable intermediate product, likely free radicals that may might attack DNA, is produced. However, the molecular basis underlying QdNO activation and QdNO-induced DNA damage has not yet been clarified.

In this study, transcriptomic and proteomic analyses were employed to examine the effects of QdNOs on susceptive *E*. *coli*. The results demonstrated that the SOS response and oxidative stress were generally induced in the bacteria exposed to QdNOs, suggesting that QdNOs killed the bacteria mainly by their induction of oxidative DNA damage. The QdNO-activating enzyme(s) and the QdNO-induced radicals were identified and the mechanism of QdNO-induced DNA damage was investigated. This study not only supports the previous findings but also provides new insights into the antibacterial mechanism of QdNOs from a systematic perspective, which will guide future research and development of this type of compounds.

## Materials and Methods

### Drugs and Chemicals

**Fig 1:**
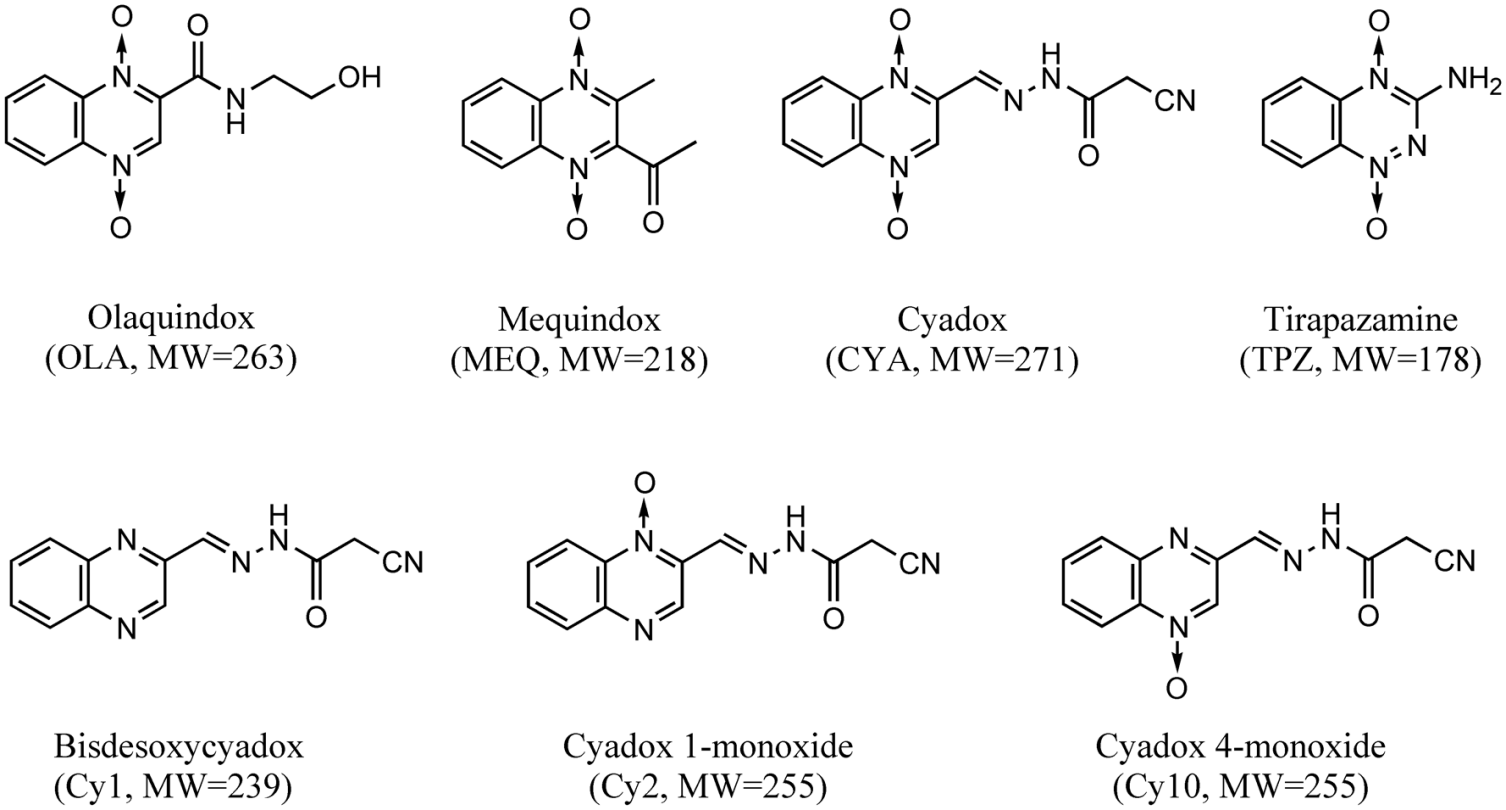
Chemical structures of QdNOs and their metabolites.

CYA, bisdesoxycyadox (Cy1), cyadox 1-monoxide (Cy2) and cyadox 4-monoxide (Cy10) (Fig 1) were synthesized by the Institute of Veterinary Pharmaceuticals (HZAU) (Wuhan, Hubei, China). OLA was obtained from the China Institute of Veterinary Drug Control (Beijing, China). MEQ was purchased from Beijing Zhongnongfa Pharmaceutical Co. Ltd. (Huanggang, Huibei, China). An ROS assay kit and dihydroethidium were purchased from Beyotime (Shenzhen, China). 3'-(*p*-hydroxyphenyl) fluorescein (HPF) and the Trizol Reagent were bought from Invitrogen (Carlsbad, CA, USA). Ciprofloxacin, carbenicillin, enrofloxacin, gentamycin, tirapazamine (TPZ), sodium 4,5-dihydroxybenzene-1,3-disulfonate (tiron), β-mercaptoethanol (β-ME), xanthine oxidase of *E*. *coli* K12, xanthine, oxypurinol, 4-methylpyrazole, and raloxifene were purchased from Sigma (St Louis, MO, USA). 5,5-Dimethyl-1-pyrroline-*N*-oxide (DMPO), *N*-tert-butyl-alpha-phenylnitrone (PBN) and 2,2-dipyridyl were obtained from Fluka (Buchs, Switzerland). Auranofin and dicoumarol were purchased from J&K Chemical Company (Shanghai, China). Oxonic acid was obtained from Tokyo Chemical Industry (Shanghai, China). Rotenone and dimercaprol were purchased from Aladdin (Shanghai, China). All other chemicals and reagents commercially available were of the highest analytical grade.

### Bacteria and plasmids

*Escherichia coli* CVCC196, *E*. *coli* CVCC216, *E*. *coli* CVCC220, *E*. *coli* CVCC223, *E*. *coli* CVCC224, *E*. *coli* CVCC1500, *E*. *coli* CVCC1502, *E*. *coli* CVCC1513, *E*. *coli* CVCC1514, *E*. *coli* CVCC1519, *E*. *coli* CVCC1496, *E*. *coli* CVCC2943, and *E*. *coli* Ae1 were obtained from the China Institute of Veterinary Drug Control (IVDC). *E*. *coli* ATCC25922 was purchased from the American Type Culture Collection (ATCC). *E*. *coli* DH5α was purchased from BestBio (Shanghai, China). Supercoiled plasmid pBR322 and pUC-19 were purchased from Takara (Dalian, China).

### Antibacterial susceptibility test

The minimum inhibitory concentration (MIC) of QdNOs against *E*. *coli* was determined by the microdilution method according to the M7-A8 CLSI standard [6]. The test was conducted under aerobic or anaerobic conditions (85% N_2_, 10% H_2_, and 5% CO_2_) at 37°C for 16 to 18 h. The MIC was defined as the minimum drug concentration in the clear wells. To determinate the minimum bactericidal concentration (MBC), 100 μl of the cultures from the clear wells in the MIC test were plated on agar broth, and the viable cells were counted. The MBC was defined as the minimum drug concentration at which 99.95% of the bacterial cells were killed.

### Scanning electron microscopy (SEM)

Bacteria were exposed to 0.5, 1 and 4 μg/ml CYA for indicated times under anaerobic conditions. The bacteria were then fixed in 2.5% glutaraldehyde at 4°C for 12 h, and then sequentially dehydrated in 20%~100% ethanol. The cells were then collected freeze-dried for 3 h, gold-plated and subjected to SEM analysis (Hitachi S-570, Tokyo, Japan).

### Microarray analysis

An overnight culture of *E*. *coli* CVCC2943 was diluted 1:100 into Luria-Bertani (LB) broth and maintained under anaerobic conditions for 1 h. After the addition of 0.5×MIC and MBC of CYA, MIC and MBC of OLA, or 1% (vol/vol) DMSO, and the cells were incubated at 37°C for 30 min. The total bacterial RNA was extracted using the Trizol reagent.

*E*. *coli* Genome 2.0 arrays (Affymetrix, Santa Clara, CA, USA) were used for hybridization. The data were analyzed using the Partek Genomics Suite (Partek, St Louis, MO, USA) and normalized with the Robust Multichip Average algorithm. Analysis of variance (ANOVA) was used to analyze the log-transformed levels. The data quality was confirmed by principal component analysis, histogram analysis and box-whisker plots. Genes associated with either >2-fold or <-2-fold changes with *P* values of less than 0.05 were considered differentially expressed genes.

The relative transcript amount was measured by quantitative reverse transcription-PCR (qRT-PCR). The primers designed for six selected genes are presented in Table 1. The fold changes of the selected genes were normalized to that of the reference 16S rRNA gene.

**Table 1:** Primer sequences for real-time PCR.

Gene	Sequence (5’-3’)		Tm (°C)
	Forward primer	Reverse primer	
*16S* *	GCTACAATGGCGCATACAAA	TTCATGGAGTCGAGTTGCAG	56
*recA*	CGGGTAACCTGAAGCAGTCCAA	GGATGTCGAGACGAACAGAGGC	65
*lexA*	ATTATGGATGGCGACTTGCTGG	CAGTTCGACTTTATTGCCCTGTTTT	65
*recN*	GTGGTGAATTGTCCCGCATCG	CCGCTAATCCCTACATCCACTTC	65
*sbmC*	GAAGGAGTGGGTTGCTGTCTATT	GAATGACGCCCTCACTGTTTT	65
*rbsC*	ACGAAAGCGTGGCTGATGGAG	AATGGCGTTCACTGAGGTTTGC	63
*treB*	GCGTTGATTAGCGGCGGTTTG	TCGTAGATCGTTTGCAGGGAAGG	56
*fliM*	CAGCGAAGTCAAAGACGAACCG	GGCAAATTCATGGTACGGCTGA	65

Note:

* Primers of 16S rRNA gene were designed according to the reference [30].

### Proteomic analysis

*E*. *coli* CVCC2943 cells were treated with 0.5×MIC/MBC of CYA or MIC/MBC of OLA at 37°C for 30 min as described above in “Microarray analysis”. The proteins of QdNO-treated *E*. *coli* were extracted mainly according to Pasquali *et al*. [7]. Two-dimensional gel electrophoresis (2-DE) was performed according to Görg *et al*. [8]. The gels were analyzed using the PDQuest software 7.0 (Bio-Rad). The protein spots that present in qualitative or quantitative differences were excised from the gels and digested in-gel with trypsin [9]. Matrix-assisted laser desorption ionization time-of-flight tandem mass spectrometry (MALDI-TOF MS/MS) was performed using a 4800 Proteomics Analyzer (Applied Biosystems, Foster City, CA, USA). The MS spectra were searched against UniProtKB/Swiss-Prot database using Mascot (Matrix Science, London, U.K.).

### Detection of free radicals from *E*. *coli* exposed to QdNOs

An overnight culture of *E*. *coli* CVCC2943 was inoculated into fresh LB medium (1:100) and incubated at 37°C for 1 h with shaking at 250 rpm. This culture was used for subsequent drug treatments. The bacterial culture was treated with 0.5×MIC, MIC and MBC of QdNOs and 0.3% (anaerobic condition) or 10% (aerobic condition) DMSO as a control. After incubation with drugs for the indicated times at 37°C, the reactions were stopped at 4°C. To detect ROS, the bacteria cells were resuspended in 500 μl of PBS containing 10 μM 2’,7’-dichlorfluorescein-diacetate (DCFH-DA) and incubated in the dark at 37°C for 40 min. The QdNO-treated bacteria were then resuspended in 350 μl of PBS and analyzed using a fluorescence microplate reader (Bio-Tek) with excitation and emission wavelengths of 490 and 530 nm, respectively. To detect O_2_^-^· radicals, the QdNO-treated bacteria (0.5 μg/ml ciprofloxacin as a positive control [10]) were resuspended in 500 μl of PBS containing 10 μM dihydroethidium and incubated in the dark at 37°C for 40 min. The cell pellet was resuspended in 1 ml of PBS for fluorescence analysis with excitation and emission wavelengths of 310 and 600 nm, respectively.

### Effects of radical scavengers and enzyme inhibitors on *E*. *coli* exposed to QdNOs

An overnight culture of *E*. *coli* CVCC2943 was inoculated into fresh LB medium (1:100) and incubated at 37°C for 1 h with shaking at 250 rpm. This culture was then used for subsequent drug treatments. The indicated free radical scavengers at a concentration of 100 mM were then added to bacterial culture, and this step was followed by the addition of 4 μg/ml CYA under anaerobic conditions. After incubation at 37°C for 3 h, the viable cell counts were calculated. Bacteria were pretreated with the O_2_^-^· scavenger tiron at a concentration of 100 mM and incubated at 37°C for 15 min. After addition of CYA and incubation at 37°C for the indicated times, the viable cells were counted.

The bacteria were incubated with 20 μM rotenone (to prevent electron transfer from the Fe-S center to ubiquinone) or 40 μM dimercaprol (to block electron transfer from cytochrome *b* to cytochrome *c*_1_) at 37°C for 0.5 h and then treated with 2×MIC of enrofloxacin, CYA or MEQ at 37°C for 45 min under anaerobic conditions to detect the level of ROS. Under aerobic conditions, the bacteria were incubated with 20 μM rotenone or 40 μM dimercaprol at 37°C for 0.5 h and then treated with MIC of enrofloxacin, CYA or MEQ at 37°C for 45 min to detect the level of O_2_^-^· radicals.

Under anaerobic conditions, the bacteria were incubated with the indicated enzyme inhibitors, including 2,2’-dipyridyl (iron chelator), oxypurinol (xanthine oxidase inhibitor), 4-methypyrazole (alcohol dehydrogenase inhibitor), dicoumarol (lipoamide dehydrogenase inhibitor), raloxifene (aldehyde dehydrogenase inhibitor), auranofin (thioredoxin inhibitor) and oxonic acid (uricase inhibitor), at a concentration of 20 μM at 37°C for 0.5 h and then treated with the MBC of CYA or MEQ at 37°C for 3 h to calculate the viable cell counts or for 0.5 h to detect the level of ROS. Under aerobic conditions, the bacteria were incubated with 20 μM of each inhibitor at 37°C for 0.5 h and then treated with 4 μg/ml CYA at 37°C for 1 h to detect the level of O_2_^-^· radicals.

### Detection of QdNO-induced DNA damage

*E*. *coli* DH5α cells were transformed with the pUC-19 or PBR322 plasmid, and the bacteria containing the plasmids were then grown in LB media at 37°C for 8 h. The culture was centrifuged at 3,000 *g* for 10 min, and the pellet was suspended in PBS and incubated with the indicated concentration of CYA or OLA or with 128 μg/ml TPZ as a positive control [11] at 37°C for 2 h under anaerobic conditions. To examine the effects of free radical scavengers on the QdNO-induced DNA damage, *E*. *coli* cells containing pUC-19 were pretreated with the indicated radical scavengers at a concentration of 100 mM. After the addition of 128 μg/ml CYA, the cells were incubated at 37°C for 2 h. The plasmids were extracted and electrophoretically separated on 1% agarose gels.

In the presence or absence of xanthine oxidase/xanthine (XO/X), pBR322 (10 μg/ml) was incubated with the indicated concentrations of QdNOs at 37°C for 0.5 h prior to electrophoresis analysis.

### Electron paramagnetic resonance (EPR) analysis

EPR was performed using a Bruker EMX-plus EPR spectrometer (Bruker, Germany) operated at 9.4 GHz. CYA or its metabolites and 50 μl of PBN or DMPO were pre-degassed separately with N_2_ gas. Two hundred microliters of CYA (1 mg/ml) was added with 2 ml of bacterial cells, extracted bacterial proteins, or XO/X respectively in tubes flushed with N_2_ and the mixtures were maintained in an oil bath at 37°C for 15 min. The EPR spectra were monitored at the following conditions: microwave frequency, 9.4 GHz; microwave power, 18 mW; modulation amplitude, 3.0 G; field sweep from 3000 to 3800 G; time constant, 80 ms; conversion time, 30 ms. The spectra were simulated using the WinEPR program (Bruker).

## Results

### The antibacterial activity of QdNOs against *E*. *coli*

*E*. *coli* strains isolated from pigs or chickens were more susceptible to QdNOs in the absence than in the presence of oxygen (Table 2), and *E*. *coli* CVCC2943, which was chosen for the subsequent omic experiments, was the most susceptible strain. CYA was metabolized into Cy1, Cy2 and Cy10 in *E*. *coli* CVCC2943 under both aerobic and anaerobic conditions (S1 Fig, see S1 Text for details of the procedure), indicating that QdNO was mainly reduced at the two *N*-oxide groups on the quinoxaline ring. The *N-*deoxy metabolites of CYA showed no antibacterial activity (MIC>128 μg/ml), indicating that the two *N*-oxide groups were necessary for the antibacterial activity of QdNOs.

**Table 2:** Antibacterial activities of CYA or OLA against *E*. *coli* strains under aerobic and anaerobic conditions.

						(μg/ml)			
*E*. *coli* strains	Serotype	CYA				OLA			
		Aerobic		Anaerobic		Aerobic		Anaerobic	
		MIC	MBC	MIC	MBC	MIC	MBC	MIC	MBC
*Control*									
ATCC25922		8	128	1	2	16	128	2	8
*Swine-origin*									
CVCC196	O8:K87, K88ac	32	64	2	8	16	128	4	8
CVCC216	O8:K87, K88ad	32	64	4	16	32	128	8	16
CVCC220	O101:K32	32	128	2	8	16	128	4	8
CVCC223	O141:K99	32	64	1	8	16	>128	2	16
CVCC224	O149:K91, K88ac	32	64	2	16	32	128	4	8
CVCC1500	O149:K88ac	32	128	4	8	32	128	8	16
CVCC1502	O9:K88	32	128	4	16	32	64	4	16
CVCC1513	O101:K99	32	>128	1	8	16	128	2	8
CVCC1514	O45:K99	32	128	0.5	16	8	16	2	8
CVCC1519	O139	32	>128	2	32	32	128	8	16
*Chicken-origin*									
CVCC1496	O139:K+	32	128	2	32	NA	NA	NA	NA
CVCC2943	O1	16	128	1	4	16	128	4	16
Ae1		32–64	128	4	32	NA	NA	NA	NA

**Fig 2:**
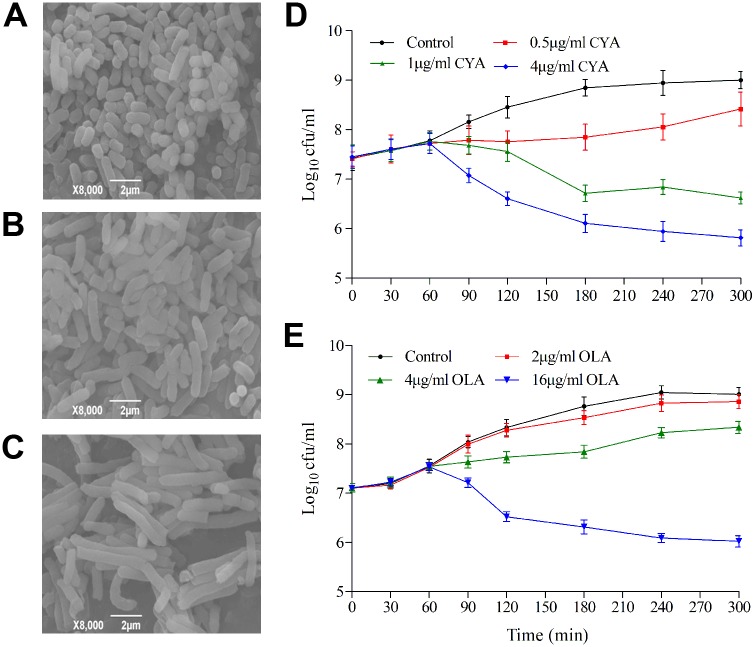
Growth of *E*. *coli* CVCC2943 exposed to QdNOs. (A-C) Morphology of *E*. *coli* CVCC2943 exposed to CYA. Bacteria were treated with 1% DMSO as a control for 24 h (A) or with 1 μg/ml CYA for 4 h (B) and 24 h (C) under anaerobic conditions. The bacterial morphology was observed by SEM as described in materials and methods. (D, E) Killing curves of *E*. *coli* CVCC2943 exposed to QdNOs. The bacteria were exposed to 0.5 (0.5×MIC), 1 (MIC), and 4 μg/ml (MBC) CYA (D), and 2 (0.5×MIC), 4 (MIC), 16 μg/ml (MBC) OLA (E), or with 1% DMSO as a control.

Bacteria exposed to CYA became elongated (Fig 2A–2C), indicating the cell division was halted. The killing of bacteria became more rapid with the increasing concentration of QdNOs (Fig 2D–2E), indicating that QdNOs are concentration-dependent antibacterials.

### Transcriptomic profiles of *E*. *coli* CVCC2943 exposed to QdNOs

The transcriptomic profiles of *E*. *coli* exposed to CYA and OLA were compared. Twenty-five genes were activated by 0.5×MIC CYA beyond the threshold levels (>2-fold change), and the *lexA*-dependent genes associated with the SOS response represented 60% of all of the up-regulated genes (Table 3). SOS genes were triggered by CYA in a dose-dependent manner, and three other SOS genes (*umuD*, *umuC*, and *ruvB*) were up-regulated by the MBC of CYA (S1 Table). More than 100 genes classified in seven functional categories were altered significantly in the OLA profiles, and approximately 20 genes were SOS genes (S1 Table).

**Table 3:** Selected differentially transcribed genes of *E*. *coli* exposed to CYA or OLA.

Gene	Relative change in gene transcription			
	CYA		OLA	
	0.5×MIC	MBC	MIC	MBC
***SOS response*** ^a^				
*c0943*	**12.13**	**44.19**	**31.84**	**62.06**
*recN*	**9.95**	**21.52**	**15.91**	**22.24**
*yebG*	**9.26**	**18.56**	**12.35**	**22.28**
*sulA*	**8.59**	**17.59**	**13.50**	**15.36**
*recA*	**7.46**	**11.42**	**9.70**	**13.88**
*yebF*	**7.22**	**20.03**	**9.17**	**15.67**
*lexA*	**4.07**	**4.54**	**4.30**	**4.68**
*dinI*	**3.86**	**5.01**	**4.57**	**5.47**
*recX*	**3.33**	**6.11**	**5.10**	**6.05**
*uvrB*	**3.18**	**5.15**	**3.05**	**6.75**
*uvrA*	**2.78**	**3.75**	**4.20**	**4.41**
*dinD*	**2.72**	**4.43**	**5.48**	**5.24**
*c3144*	**2.52**	**8.71**	**6.82**	**11.16**
*dinF*	**2.48**	**2.44**	**2.58**	**2.49**
*ydjQ*	**2.41**	**2.64**	**2.98**	**2.22**
*ruvA*	**2.39**	**2.43**	**2.30**	**2.43**
*ydjM*	**2.33**	**2.46**	**2.56**	**2.31**
*sbmC*	**2.24**	**2.74**	**4.27**	**2.91**
*polB*	**2.07**	**2.45**	**2.79**	**2.96**
***Other stress responses*** ^b^				
*yjbJ*	**2.47**	**3.11**	1.69	**2.88**
*intT*	**2.32**	**5.31**	**4.66**	**6.27**
*yafO*	1.**96**	**2.26**	**2.10**	**2.51**
*smpA*	1.77	**2.52**	**2.21**	**2.94**
*ydhC*	1.61	**2.89**	1.52	**2.10**
*marA*	1.30	**2.02**	1.01	**3.01**
*yfaE*	1.25	**2.41**	**2.54**	**2.84**
*bhsA*	1.10	1.97	1.67	**2.43**
*gyrA*	1.30	1.79	1.57	1.73
*gyrB*	1.10	1.53	1.12	1.76
*proV*	1.05	**-2.04**	1.06	**-2.15**
*treB*	-1.06	**-2.80**	-1.54	**-2.82**
*fliM*	-1.12	**-2.21**	-1.89	**-2.07**
*treC*	-1.13	**-2.56**	-1.59	**-2.55**
*rbsC*	-1.14	**-2.41**	-1.40	**-2.86**
*hyaE*	-1.76	**-2.15**	-1.77	**-2.43**
*napF*	-1.78	**-2.12**	**-3.22**	**-3.43**

Numbers in bold indicate a 2>-fold change or <-2-fold change. 0.5×MIC, MIC, and MBC indicate drug concentrations.

^a^ DNA damage response genes were selected with a >2-fold change in all drug treatment groups.

^b^ Stress response genes were selected as the consistently up- or down-regulated in a dose-dependent manner for both drugs.

The transcriptions of stress response genes was also changed in the QdNO profiles (Table 3). *marA* encoding a DNA-binding transcriptional regulator was up-regulated. *yfaE* involved in the synthesis of ribonucleoside diphosphate reductase was up-regulated, whereas *treB* and *treC*, which are involved in the metabolism of carbohydrate or amino acids, were down-regulated. *napF*, which participates in the activation of periplasmic nitrate reductase, and *hyaE*, which encodes the hydrogenase isoenzyme 1 chaperone, were down-regulated. Among the cell envelope genes, *proV* and *rbsC* encoding membrane transporters were down-regulated, whereas *bhsA* and *smpA*, which encode outer membrane proteins, were up-regulated. Among the virulence genes, *fliM* encoding a flagella protein was down-regulated. In addition, *yjbJ*, which is involved in osmotic stress, and *intT* encoding a prophage integrase were up-regulated. In the OLA profile, *mdaB*, *dps*, *hdeA* and *sodB*, which are involved in the oxidative stress resistance, *bsmA* and *bhsA*, which are involved in peroxide resistance in biofilm, and *sdhC*, *sdhD*, and *cyoABC*, which are involved in oxidative phosphorylation were up-regulated (S1 Table).

Four induced SOS genes (*recA*, *recN*, *sbmC* and *lexA*) and three down-regulated genes (*fliM*, *rbsC* and *treB*) were verified by qRT-PCR (Table 4).

**Table 4:** Validation of gene transcription profiles by real-time PCR.

Expression pattern	Gene	Ratio by microarray analysis				Ratio by RT-PCR			
		0.5×MIC CYA	MBC CYA	MIC OLA	MBC OLA	0.5×MIC CYA	MBC CYA	MIC OLA	MBC OLA
Conserved	*rrn*	0.96	0.96	0.96	0.92	1	1	1	1
Up-regulated	*recA*	7.46	11.42	9.70	13.88	5.97	12.41	8.60	19.05
	*lexA*	4.07	4.54	4.30	4.68	2.62	2.53	3.18	2.29
	*recN*	9.95	21.52	15.91	22.24	5.85	9.02	6.03	8.45
	*sbmC*	2.24	2.74	4.27	2.91	2.54	2.70	2.38	2.17
Down-regulated	*rbsC*	0.88	0.41	0.72	0.35	1.22	0.49	0.85	0.49
	*treB*	0.94	0.36	0.65	0.35	1.16	0.40	0.83	0.32
	*fliM*	0.89	0.45	0.53	0.48	1.29	0.46	0.79	0.49

0.5×MIC, MIC, and MBC indicate drug concentrations.

### Proteomic profiles of *E*. *coli* CVCC2943 exposed to QdNOs

In pH 3–10 gels, differential proteins were observed only in the CYA groups (S2 Fig, S2 Table). The DNA protection-related protein Dps was significantly up-regulated. A bifunctional protein (FolD), a NADH-quinone oxidoreductase subunit (NuoC/D), the single-stranded DNA-binding protein (Ssb) and the respiration nitrate reductase beta subunit (NarY) appeared. In the pH 4–7 gels, over twenty protein spots showed altered expression (S2 Fig, S3 Table). Elongation factor Tu1 (EftU1) was absent or down-regulated significantly in QdNO profiles.

The synthesis rates of some proteins (e.g., EftU1, NuoC/D, YgfZ, PflB, NanE, IadA, NarY, and FolD) were markedly more pronounced than those of their corresponding mRNAs. LexA repressor was down-regulated at the protein level but significantly up-regulated at the mRNA level because LexA was auto-digested during SOS response.

### QdNO induced free radicals in *E*. *coli*

**Fig 3:**
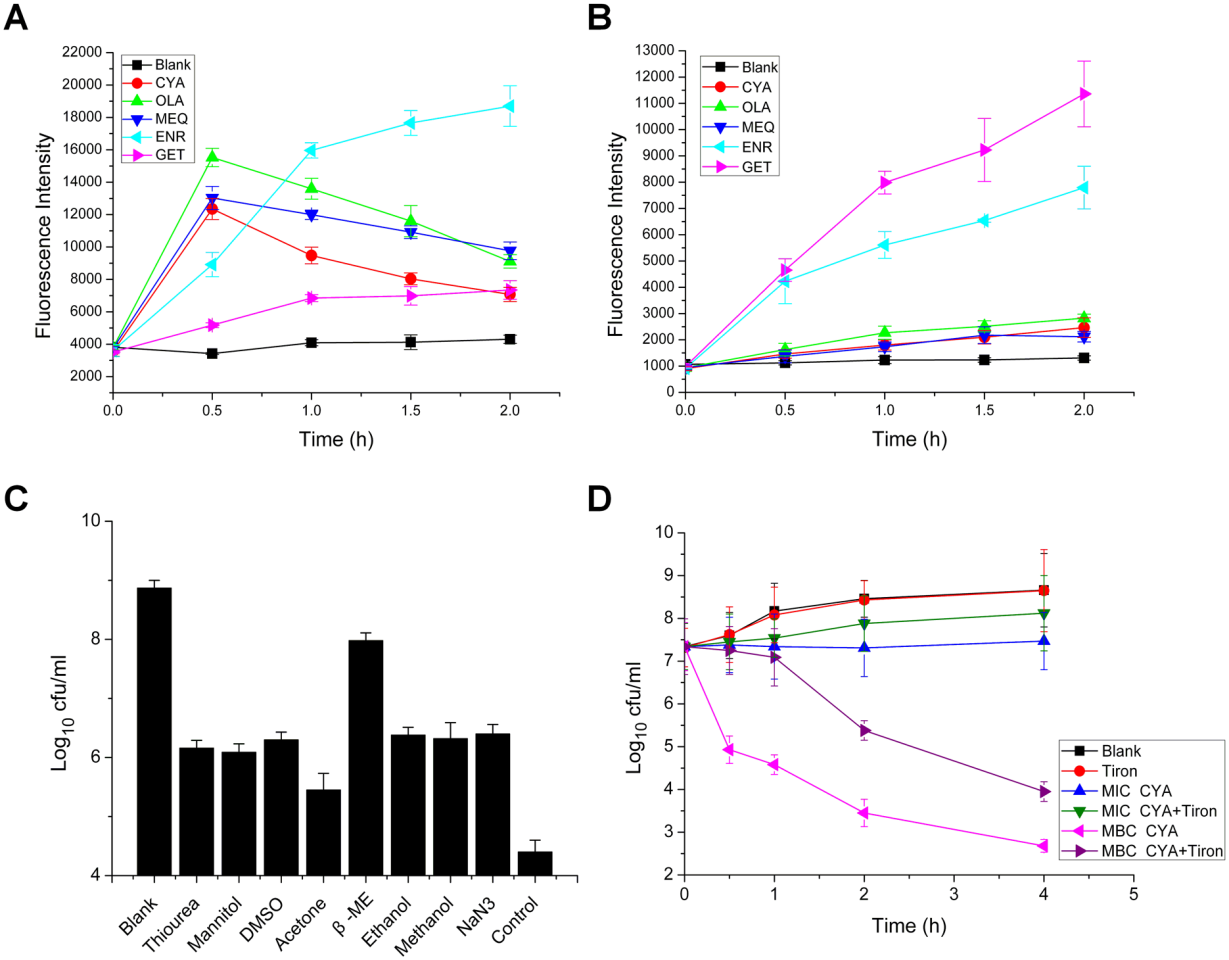
Intracellular free radical levels (A, B) and effects of free radical scavengers (C, D) on *E*. *coli* CVCC2943 exposed to QdNOs. (A) Under anaerobic conditions, the bacteria were treated with the MBC of CYA (4 μg/ml), OLA (16 μg/ml), MEQ (2 μg/ml), enrofloxacin (0.03 μg/ml), and gentamycin (2 μg/ml). After incubation for the indicated times, the level of ROS was detected. (B) Under aerobic conditions, *E*. *coli* CVCC2943 cells were treated with 2×MIC of CYA (32 μg/ml), OLA (32 μg/ml), MEQ (4 μg/ml), enrofloxacin (0.03 μg/ml), and gentamycin (2 μg/ml). After incubation of bacteria with the drugs for the indicated times, the level of O_2_^-^· radicals was detected. (C) Under anaerobic conditions, the bacteria were pretreated with the indicated free radical scavengers at a concentration of 100 mM, and CYA at the MBC (4 μg/ml) was then added. Samples were collected 3 h after addition of the drug, and the surviving bacteria were counted. (D) Under aerobic conditions, bacteria were pretreated or not pretreated with tiron before the addition of indicated concentration of CYA. At the indicated times, the surviving bacteria were counted. The data are presented as the means ± SDs (error bars), n = 3. ENR, enrofloxacin; GET, gentamycin.

Under anaerobic conditions, ROS were detected in bacteria exposed to QdNOs (Fig 3A). Different from enrofloxacin and gentamycin, which push cellular respiration into overdrive to produce dangerous amounts of ROS [10], the amount of ROS induced by QdNOs reached the highest level at 0.5 h but declined afterwards, indicating that ROS may be obtained from the *N*-deoxy metabolism of QdNOs. Under aerobic conditions, QdNOs induced a release of O_2_^-^· radicals in bacteria (Fig 3B). The production of ROS and O_2_^-^· radicals increased with an increase in the concentration of QdNOs (S3A Fig). No O_2_^-^· radicals were observed for the *N*-deoxy metabolites (S3B Fig), indicating that the reductive activation of QdNO is involved in O_2_^-^· formation. ·OH radicals were not detected in bacteria exposed to CYA under anaerobic conditions, whereas carbenicillin induced the production of ·OH radicals, as previously reported [10] (S4 Fig, see S1 Text for details of the procedure).

Under anaerobic conditions, eight types of free radical scavengers could inhibit the antibacterial activity of CYA to certain extents (Fig 3C), indicating that the free radicals play an important role in the antibacterial action of CYA. In particular, β-ME and NaN_3_ presented the two highest inhibition effects. The O_2_^-^· scavenger tiron partially inhibited the antibacterial activity of CYA (Fig 3D), suggesting that O_2_^-^· radicals are involved in the antibacterial action of CYA under aerobic condition.

### Origin of QdNO-induced radicals in *E*. *coli*

**Fig 4:**
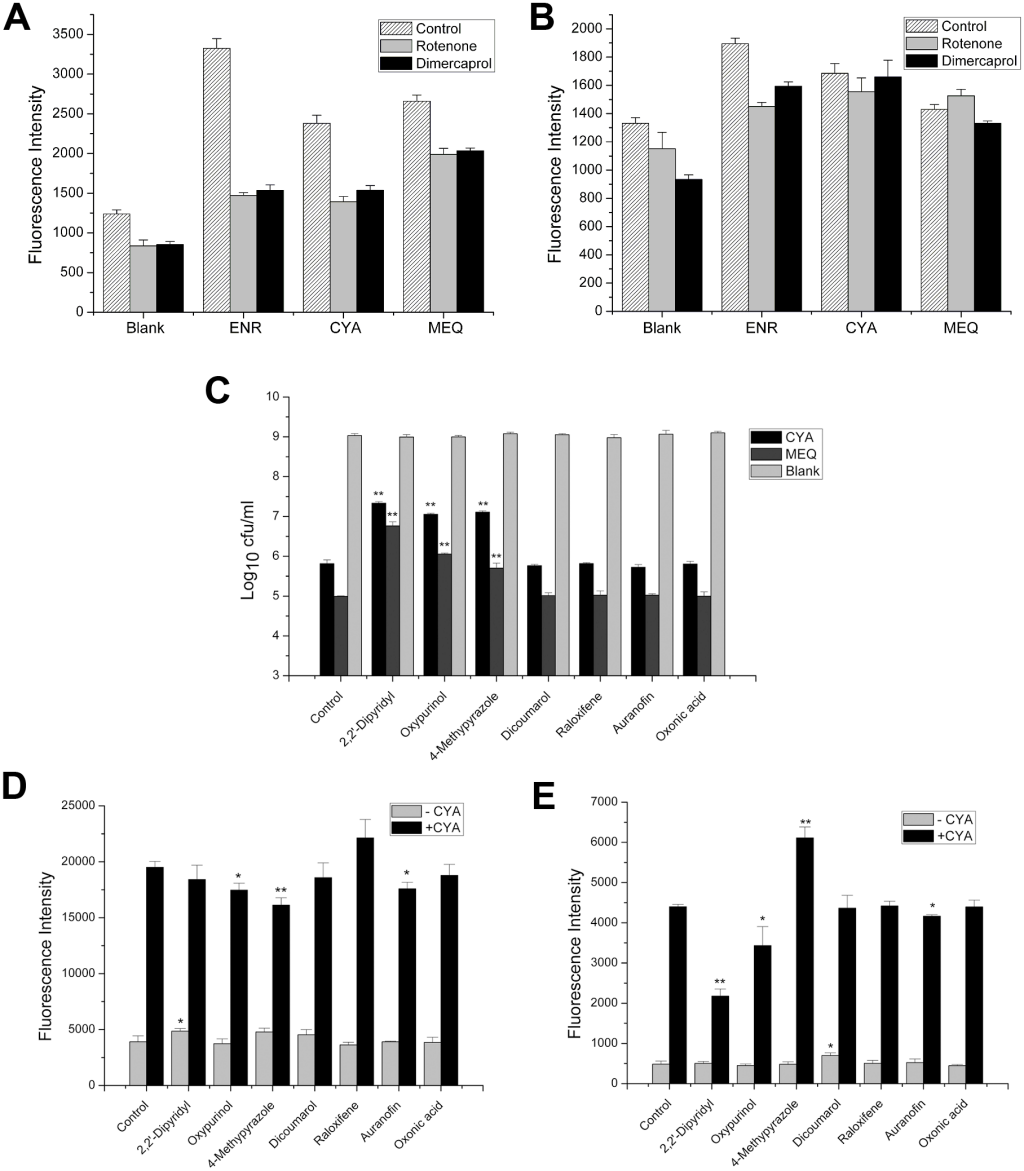
Effects of electron transfer chain inhibitors (A, B) and enzyme inhibitors (C-E) on the generation of ROS and O_2_^-^· and the growth of *E*. *coli* CVCC2943 exposed to QdNOs. (A) Under anaerobic conditions, the bacteria were incubated with 20 μM rotenone or 40 μM dimercaprol for 0.5 h and then treated with 2×MIC of enrofloxacin (ENR, 0.03 μg/ml), CYA (2 μg/ml) and MEQ (4 μg/ml) for 45 min. The level of ROS was detected as described in materials and methods. (B) Under aerobic condition, the bacteria were incubated with 20 μM rotenone or 40 μM dimercaprol for 0.5 h and then treated with the MIC of enrofloxacin (0.015 μg/ml), CYA (16 μg/ml), and MEQ (2 μg/ml) for 45 min. The level of O_2_^-^· radicals was detected as described in materials and methods. All of the experiments were performed at 37°C. The data are presented as the means ± SDs (error bars), n = 3. (C) Under anaerobic conditions, the bacteria were incubated with the indicated inhibitor at a concentration of 20 μM for 0.5 h and then treated with the MBC of CYA (4 μg/ml) or MEQ (2 μg/ml) for 3 h. The viable cell counts were calculated by the agar dilution plate count method. (D) Under anaerobic conditions, the bacteria were incubated with each inhibitor at a concentration of 20 μM for 0.5 h and were then treated with 4 μg/ml CYA for 0.5 h. The level of ROS was detected. (E) Under aerobic conditions, the bacteria were incubated with each inhibitor at a concentration of 20 μM for 0.5 h and then treated with 4 μg/ml CYA for 1 h. The level of O_2_^-^· radicals was detected. The data were presented as the means ± SDs (error bars), n = 3.

The expression of some genes and proteins involved in the TCA cycle and respiration were altered in the QdNO profiles (Table 5), thus whether the electron transfer chain was interfered by QdNOs was investigated using specific inhibitors. Under anaerobic condition, rotenone and dimercaprol inhibited the production of ROS in all drug-treated groups, but the inhibition ratios of QdNO groups were less than that of the enrofloxacin group (Fig 4A), indicating that the QdNO-induced ROS came from other origin(s) except for respiration-origin. Under aerobic conditions, inconsistent with the enrofloxacin group, the two inhibitors could not alleviate the generation of O_2_^-^· radicals in the QdNO groups (Fig 4B), indicating that most of the O_2_^-^· radicals were yielded from the metabolism of QdNOs.

**Table 5:** Comparison between the synthesis rates of the proteins and the corresponding mRNAs of *E*. *coli* exposed to CYA or OLA.

Protein name	Gene name	Fold change mRNA (Protein)			
		0.5×MIC CYA	MBC CYA	MIC OLA	MBC OLA
FumA	*fumA*	-1.10	-1.38	1.93	1.40 (<-2)
MreB	*mreB*	1.11 (Absent)^a^	1.33	1.20	1.46
SelB	*selB*	1.01	1.20	1.13	1.30 (<-2)
EftU1	*tuf*	1.02 (Absent)	1.00 (<-2)^b^	1.01 (<-2)	1.08
KpsU	*kpsU*	-1.23	-1.10	-1.18	-1.03 (<-2)
Ssb	*ssb*	1.46	1.86 (Appear)^c^	1.56	1.74 (>2)^d^
YfbU	*yfbU*	1.07 (<-2)	1.05	1.22 (<-2)	1.35
CH60	*groL*	1.30	1.59	1.32	1.94 (Absent)
YgfZ	*ygfZ*	1.05	1.09	-1.09	1.12 (Appear)
RpoC	*rpoC*	1.25 (Absent)	1.28	1.40	1.57
Odp1	*aceE*	1.00 (<-2)	1.11	1.42	1.23
PflB	*pflB*	1.04 (Absent)	-1.04	-1.10	1.08
NanE	*nanE*	-1.23	-1.11	-1.07 (Absent)	-1.08
IadA	*iadA*	-1.02	1.02	-1.06 (<-2)	-1.09
NarY	*narY*	1.07	1.12 (Appear)	-1.01	1.12
Dps	*dps*	1.25 (>2)	1.38	1.74	2.13
FolD	*folD*	1.01 (Appear)	1.01	1.15	1.18
NuoC/D	*nuoC*	-1.04 (Appear)	-1.04(Appear)	1.06	-1.09
LexA	*lexA*	4.07	4.54 (Absent)	4.30	4.68(< -2)

^a^ Absent represents proteins that only appear in control group.

^b^ <-2 represents proteins that are down-regulated more than 2-fold with statistical significance (*P*<0.01).

^c^ Appear represents proteins that only appear in drug-treated groups.

^d^ >2 represents proteins that are up-regulated more than 2-fold with statistical significance (*P*<0.01).

The enzyme inhibitors 2,2’-dipyridyl, oxypurinol and 4-methypyrazole impaired the antibacterial potency of QdNOs (Fig 4C). Oxypurinol, 4-methylpyrazole and auranofin decreased the formation of ROS in the CYA-treated bacteria (Fig 4D), and 2,2’-dipyridyl, oxypurinol and auranofin decreased the formation of O_2_^-^· radicals (Fig 4E). Oxypurinol could reduce both free radicals and alleviate the antibacterial effect of QdNOs, suggesting that xanthine oxidase is one of the QdNO-activating enzymes.

### QdNO-induced DNA damage

**Fig 5:**
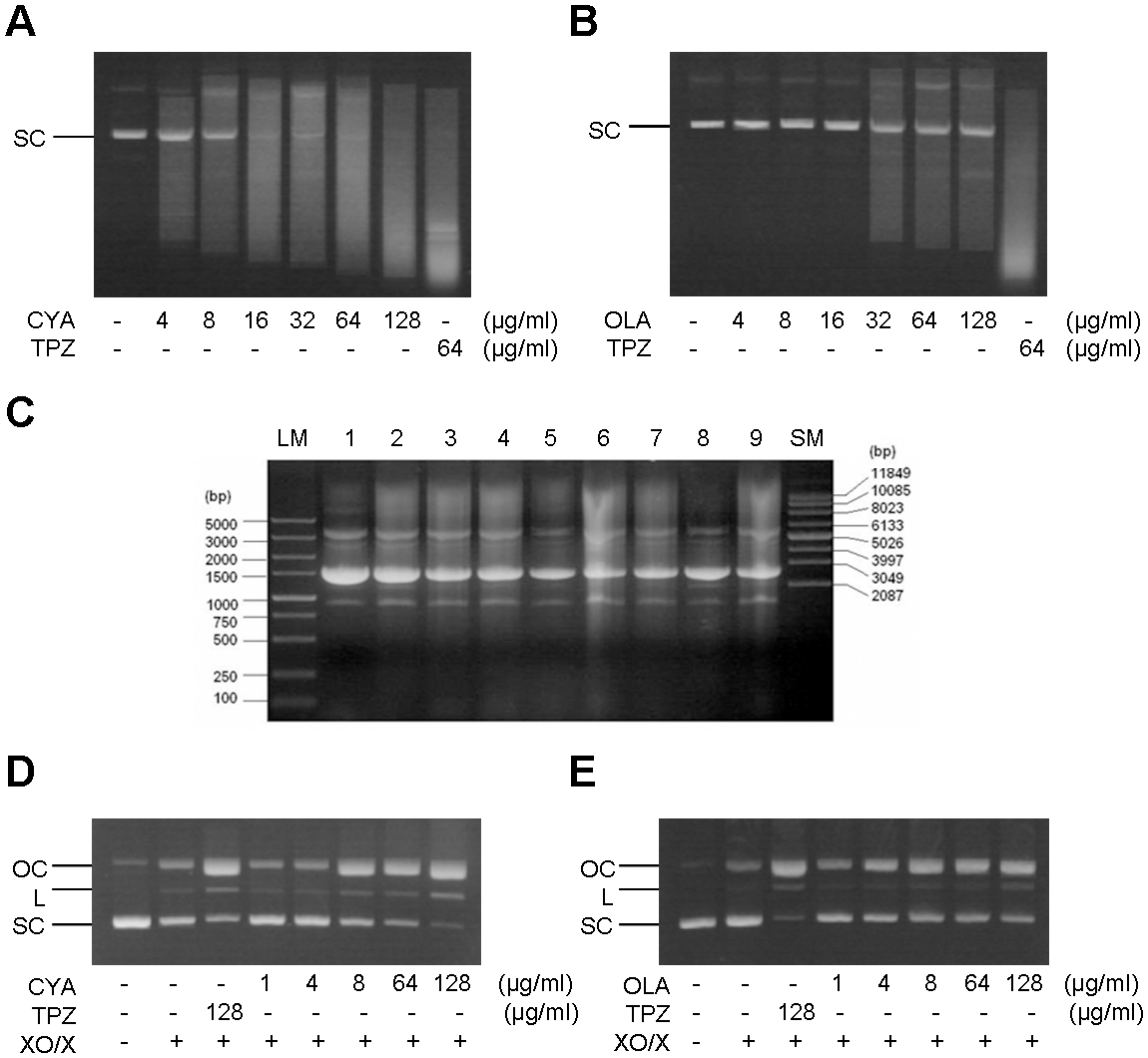
QdNO-induced DNA strand breakage. (A, B) *E*. *coli* DH5α cells harboring the pBR322 plasmid were incubated with the indicated concentration of CYA (A) or OLA (B) at 37°C for 2 h. TPZ was used as the positive control drug. (C) Inhibition of the degradation of pUC-19 plasmid in *E*. *coli* DH5α by free radical scavengers. Bacteria harboring pUC-19 plasmid were incubated with 100 mM mannitol, thiourea, acetone, β-ME, ethanol, methanol and NaN_3_ (from lane 2 to lane 8), and after 4 μg/ml CYA was added, the cells were incubated at 37°C for 2 h. The untreated bacteria (lane 1) and the bacteria treated only with CYA but no scavengers (lane 9) were used as the blank and the control, respectively. (D, E) Supercoiled pBR322 DNA (10 μg/ml) was incubated with the indicated concentration of CYA (D) or OLA (E) in the presence of xanthine oxidase/xanthine at 37°C for 0.5 h. The plasmids isolated from the treated bacteria (A-C) or the treated plasmids (D, E) were electrophoretically separated. XO/X, xanthine oxidase/xanthine; LM, linear DNA marker; SM, supercoiled DNA marker. SC, L and OC indicate supercoiled, linear and open circular DNA, respectively.

The plasmid DNA was degraded in bacteria treated with QdNOs, and the degradation level increased with the increases in the drug concentration (Fig 4A and 4B). Specifically, CYA, even at low concentration of 4 μg/ml, caused a significant degradation of plasmid DNA, wheras OLA only caused obvious DNA degradation at a concentration of 32 μg/ml, consistent with the more effective antibacterial activity of CYA compared with that of OLA. The use of radical scavengers attenuated the degradation of plasmid DNA to certain extent (Fig 5C), suggesting that the observed QdNO-induced DNA damage is related to the yield of free radicals. β-ME and NaN_3_ inhibited the plasmid degradation at the highest level, consistent with the highest inhibition effects of these two scavengers on the antibacterial activity of CYA (Fig 3C).

The prototypes of QdNOs could not damage plasmid DNA under either aerobic or anaerobic conditions (S5 Fig). However, in the presence of XO/X, QdNOs could cleave the supercoiled DNA into linear and open circular forms in a concentration-dependent manner (Fig 5). Increasing concentration of CYA also revealed a slight blueshift of the DNA maximum absorption and a hyperchromic effect in the presence of XO/X (S6 Fig), indicating that DNA was inter-cleaved by the activated CYA.

### Identification of the QdNO radicals

**Fig 6:**
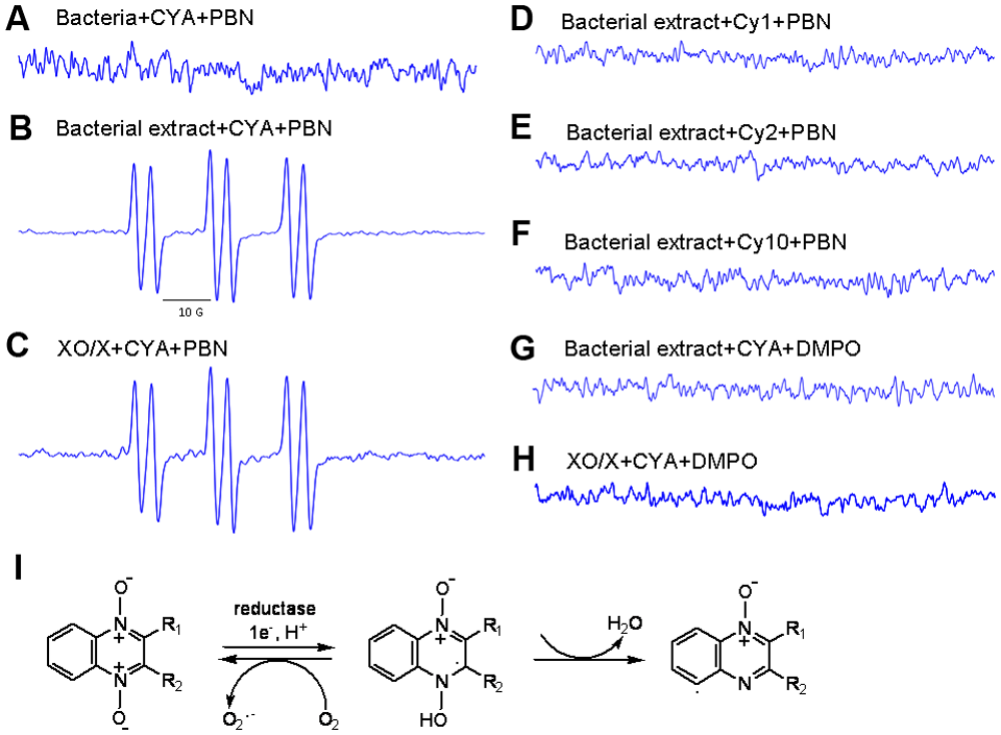
EPR spectra of radicals obtained after the reduction of CYA or its *N*-deoxy metabolites by different reductives. XO/X, xanthine oxidase/xanthine; DMPO, 5,5-dimethyl-1-pyrroline-*N*-Oxide; PBN, *N*-tert-butyl-alpha-phenylnitrone.

A doublet of triplets spectum, which is a typical spectum of PBN-carbon radical adduct (PBN-C-R adduct), was trapped from CYA incubated with the bacterial protein extract (Fig 6B). Interestingly, no species were observed from the incubation of CYA with the intact bacteria cells (Fig 6A), probably due to the short-life of the radicals formed inside the cells. The *N*-deoxy metabolites induced no radical signals (Fig 6D–6F), indicating that the unstable radicals are the active intermediates from the reduction of QdNOs.

XO/X could induce the generation of radicals in the presence of CYA (Fig 6C), and the EPR spectrum resembled to that obtained for the bacterial extract group (Fig 6B), confirming that xanthine oxidase is a QdNO-activating reductase (Fig 6I). No radicals were trapped by DMPO from the mixture of CYA with either the bacterial extract or XO/X (Fig 6G and 6H), probably because most QdNO radicals were C-centered radicals rather than oxygen-centered radicals.

## Discussion

The induction of SOS genes was the primary response of *E*. *coli* to QdNOs, indicating that QdNO are DNA-damaging agents. This is consistent with the previous findings reported by Suter *et al*. [4], who found that mutants of SOS response-related genes, including *recABC* and *uvrA*, were more susceptible to QdNO than wild type *E*. *coli*. Moreover, the profile of QdNO is similar to that of the DNA gyrase inhibitor, norfloxacin [12]. The QdNO analogue, TPZ, was found to target eukaryotic Topoisomerase II [13]. Although *gyrA* and *gyrB* were not significantly changed in the QdNO profiles, these two genes were still up-regulated in a dose-dependent manner (Table 3). Moreover, *sbmC*, encoding theDNA gyrase inhibitor GyrI which protects bacteria against fluoroquinolones [14], was up-regulated in the QdNO profiles (Table 3). Because *gyrA* and *gyrB* were only significantly up-regulated with ≥33×MIC of norfloxacin [12], the possibility that DNA gyrase is a target of QdNO cannot be excluded. Moreover, the QdNO profile is similar to the UV profile [15], suggesting that QdNOs can cause unspecific and universal DNA damage.

QdNOs also caused oxidative stress in bacteria. *marA*, which controls the global responses to different stresses including oxidative agents and antibiotics, was induced. Dps, a DNA-binding protein involved in protecting DNA against oxidative stresses, was up-regulated because QdNOs can induce the production of ROS in *E*. *coli* (Fig 3). The level of oxidative stress in the OLA profile was more significant than that observed in CYA profile (S1 Table). Our recent investigation revealed that the faster deoxy rate of QdNOs would result in a lower oxidative toxicity of QdNOs in the liver microsome [16], and CYA had faster deoxy rate than OLA. Therefore, we attributed the difference in the levels of oxidative stress between CYA and OLA to their different chemical properties.

A previous study demonstrated that bactericidal antibiotics kill bacteria by activating a common pathway involving respiration, the TCA cycle, NADH depletion, and the iron-catalyzed Fenton reaction [10]. Odp1, PflB, and FumA, which are involved in the TCA cycle, were interfered in the QdNO profiles (Table 5). NuoC/D, a component of NADH dehydrogenase I, was induced, and it is found that NADH-coupled electron transport is commonly up-regulated in response to bactericidal drugs [10]. The up-regulation of NarY and *yfaE*, which contain iron-sulfur (Fe-S) clusters, and YgfZ, which is involved in the synthesis of Fe-S clusters, likely compensate the oxidatively destabilized Fe-S clusters resulting in the Fenton reaction. However, no ·OH radicals were detected in QdNO-treated bacteria (S4 Fig), probably due to the anaerobic condition used for drug treatment because ROS are generated in proportion to oxygen concentration [17]. The radicals that originated from the interference of the TCA cycle and respiration of bacteria only corresponded to a very small propotion of the QdNO-induced radicals (Fig 4A and 4B), unlike those obtained with other bactericidal antibiotics (e.g., fluoroquinolones) [10], indicating that the radicals produced from the reduction of QdNOs contributed to most of the ROS observed. This is consistent with the results of previous study, which showed that free radicals were generated during the reduction of quindoxin [4].

Other stress response genes were also interfered by QdNOs. The induced *yafO* belongs to the YafO-YafN toxin-antitoxin system, which causes inhibition of cell growth [18]. Moreover, MreB, which is involved in the formation of the normal cellular shape, was absent in the CYA group. The elongation factor EftU1, the translation terminator SelB and the transcription related protein RpoC were either absent or down-regulated, likely in synergy with the up-regulation of the translation inhibitor gene, *yafO* and *yfiA* (S1 Table) to adapt to environmental stress. *marA*, encoding the “multiple antibiotic resistance”-activating protein [19], was up-regulated, which may be related to the up-regulation of *ydhC*, which encodes a multidrug-efflux transporter [20] (Table 3). In contrast, *rbsC*, *proV* and *treB*/*treC*, which encode transmembrane transporters, were down-regulated, probably decreasing the entry of QdNOs. The induced *smpA* is a lipoprotein in the outer membrane of *E*. *coli*, and the deletion of *smpA* results in increased sensitivity to rifampin [21].

The analysis of bacterial virulent components revealed that *fliM*, which encodes a flagellar switch protein, was down-regulated. It has been suggested that motile bacteria first induce the chemotactic response away from unfavorable conditions, and if this failed, the bacteria will shut down their flagella-protein synthesis, which saves more energy for survival [22]. In addition, *fliL* and *fliF*, involved in flagella assembly (S1 Table), and KpsU5, involved in capsular polysaccharide biosynthesis, were also down-regulated. All of these results suggest that the resistance of *E*. *coli* exposed to QdNOs was enhanced and that the virulence was reduced.

A previous study suggested that QdNOs are reductive activated by some unknown bacterial enzyme(s) [4]. This study found that xanthine oxidase is one of the QdNO-activating enzymes. Iron-containing enzymes and alcohol dehydrogenase may also play partial roles in activating QdNOs since 2,2’-dipyridyl and 4-methypyrazole could increase the survival of bacteria exposed to QdNOs. Many oxidoreductases contain a Fe-S cluster as the electron deliver unit. TPZ can be metabolized by numerous enzymes (e.g., xanthine oxidase, cytochrome P450, DT-diaphorase, and NADPH:cytochrome P450 reductase) [23–26], but DNA breakage is only caused by the reductase(s) in the nucleus [27]. Because bacterial DNA is exposed in the cytosol, it is likely that more than one bacterial reductase is involved in the QdNO-induced DNA damage. The identification and characterization of QdNO-activating enzymes may aid the development and design of some new QdNOs based on the drug-target enzyme interaction.

No ·OH radicals were detected in *E*. *coli* exposed to QdNOs. Recent studies of TPZ demonstrated that no ·OH radicals but some TPZ radicals (aminyl or phenyl radicals) were eliminated from TPZ under enzymatic reduction conditions [28, 29]. The EPR spectrum of PBN-C-R adducts from CYA demonstrated that the structures of QdNO radicals are similar to those of TPZ radicals [29], consisting of a protonated carbon-centered radical and a dehydrated aryl-type radical (Fig 6). Among various free radical scavengers, β-ME, which can compete with the action of QdNO at the C1’-position of deoxyriboses of DNA, and NaN_3_, which is a specific aromatic hydrocarbon radical scavenger, inhibited the degradation of DNA at the highest level in bacteria (Fig 5C). Therefore, the carbon-centered and aromatic QdNO radicals probably act at the C1’ position of DNA deoxyribose. Because the radical scavengers only partially inhibit the lethal action of QdNOs, there may be other pathways of QdNO-induced cell death which still need to be investigated.

In conclusion, QdNOs are redox-activated, hypoxia-selective DNA-cleaving agents. The oxidative DNA-damaging effects of QdNOs will stimulate the SOS response, oxidative stress and other protection strategies in bacteria. Therefore, one may be able to enhance the antibacterial efficacy of QdNOs by impairing DNA repair and oxidative defenses of bacteria.

## Supporting Information

S1 Fig The total ion current (TIC) chromatogram of CYA metabolites in *E*. *coli* CVCC2943 under aerobic (A) and anaerobic conditions (B). *E*. *coli* CVCC2943 cells were incubated with 4 μg/ml CYA under aerobic (A) and anaerobic conditions (B) for 0.5 h. The extracts of the metabolites from the bacteria were subjected to HPLC/ESI-IT-TOF MS as described in materials and methods.
(TIF)

S2 Fig 2-DE analysis of the protein expression in *E*. *coli* CVCC2943 exposed to CYA and OLA. (A) Distribution of the differentially expressed proteins of *E*. *coli* CVCC2943 treated with CYA and OLA in the pH 3–10 and pH 4–7 2-D gels. (B) The differentially expressed protein No. 8005 (EftU1) was present in three replicated 2-D gels from the control group and the group treated with the MBC of OLA. Protein No. 8005 was down-regulated with 99% statistical significance.
(TIF)

S3 Fig Intracellular free radical levels in *E*. *coli* CVCC2943 treated with CYA or its metabolites. (A) Under anaerobic condition, *E*. *coli* CVCC2943 cells were treated with the indicated concentration of CYA, and 0.3% DMSO was used as a blank. After incubation for the indicated times, the level of ROS was detected as described in materials and methods. (B) Under aerobic conditions, the bacteria were treated with the indicated concentration of CYA, and 10% DMSO was used as a blank. After incubation of the bacteria with drugs for the indicated times, the superoxide radial levels were detected as described in materials and methods. The fluorescence intensity ratio was calculated as the fluorescence intensity of the drug-treated sample to the fluorescence intensity of the blank sample. The data were presented as the means ± SDs (error bars), n = 3.
(TIF)

S4 Fig OH levels in *E*. *coli* CVCC2943 exposed to CYA under anaerobic conditions. Bacteria were treated with 0.5, 1, and 4 μg/ml CYA or with 0.3% DMSO as a negative control for 0.5 h (B), 1 h (C), 2 h (D) and 4 h (E) under anaerobic conditions. The bacteria were exposed to 5 μg/ml carbenicillin as a positive control for 0, 1, 2 and 4 h, respectively (A). The levels of ·OH radicals were detected as described in materials and methods.
(TIF)

S5 Fig Effects of CYA or OLA prototypes on plasmid pBR322 DNA. Supercoiled pBR322 DNA (10 μg/ml) was incubated with the indicated concentration of CYA (A, C, and E) or OLA (B, D, and F) at 37°C for 0.5 h (A-D) or the indicated times (E, F) under anaerobic or aerobic conditions. The treated plasmids were electrophoretically separated as described in materials and methods. H_2_O_2_ was set as a positive control. SC, L and OC indicate supercoiled, linear and open circular DNA, respectively.
(TIF)

S6 Fig UV absorption spectrum of DNA treated with CYA prototype (A) and CYA in the presence of XO/X (B). (A) 50 μg/ml DNA was incubated with CYA at a concentration of 0 μg/ml (black, the maximum UV absorption wavelength was located at 260 nm), 0.25 μg/ml (red, 258 nm), 0.5 μg/ml (blue, 258 nm), 1 μg/ml (cyan, 258 nm), and 2 μg/ml (magenta, 258 nm). The spectrum of 2 μg/ml CYA is indicated by a green line (298 nm). (B) 50 μg/ml DNA was incubated with CYA at a concentration of 0 μg/ml (black, 259 nm), 0.25 μg/ml (red, 254 nm), 0.5 μg/ml (blue, 253 nm), 1 μg/ml (cyan, 252 nm), and 2 μg/ml (green, 251 nm) in the presence of XO/X. The spectrum of CYA is indicated by a magenta line (298 nm).
(TIF)

S1 Table Differentially expressed genes in *E*. *coli* CVCC2943 in response to cyadox and olaquindox. (DOC)

S2 Table Differentially expressed proteins in *E*. *coli* CVCC2943 in response to CYA, as detected in a pH 3–10 2-D gel. (DOC)

S3 Table Differentially expressed proteins in *E*. *coli* CVCC2943 in response to CYA and OLA, as detected in a pH 4–7 2-D gel. (DOC)

S1 Text Supplementary materials and methods. (DOC)
