# Supplementary figures and images for: Systematic and Molecular Basis of the Antibacterial Action of Quinoxaline 1,4-Di-N-Oxides against Escherichia coli
Source: PLoS One. 2015 Aug 21;10(8):e0136450. doi: 10.1371/journal.pone.0136450 (PMC4546592; doi:10.1371/journal.pone.0136450)

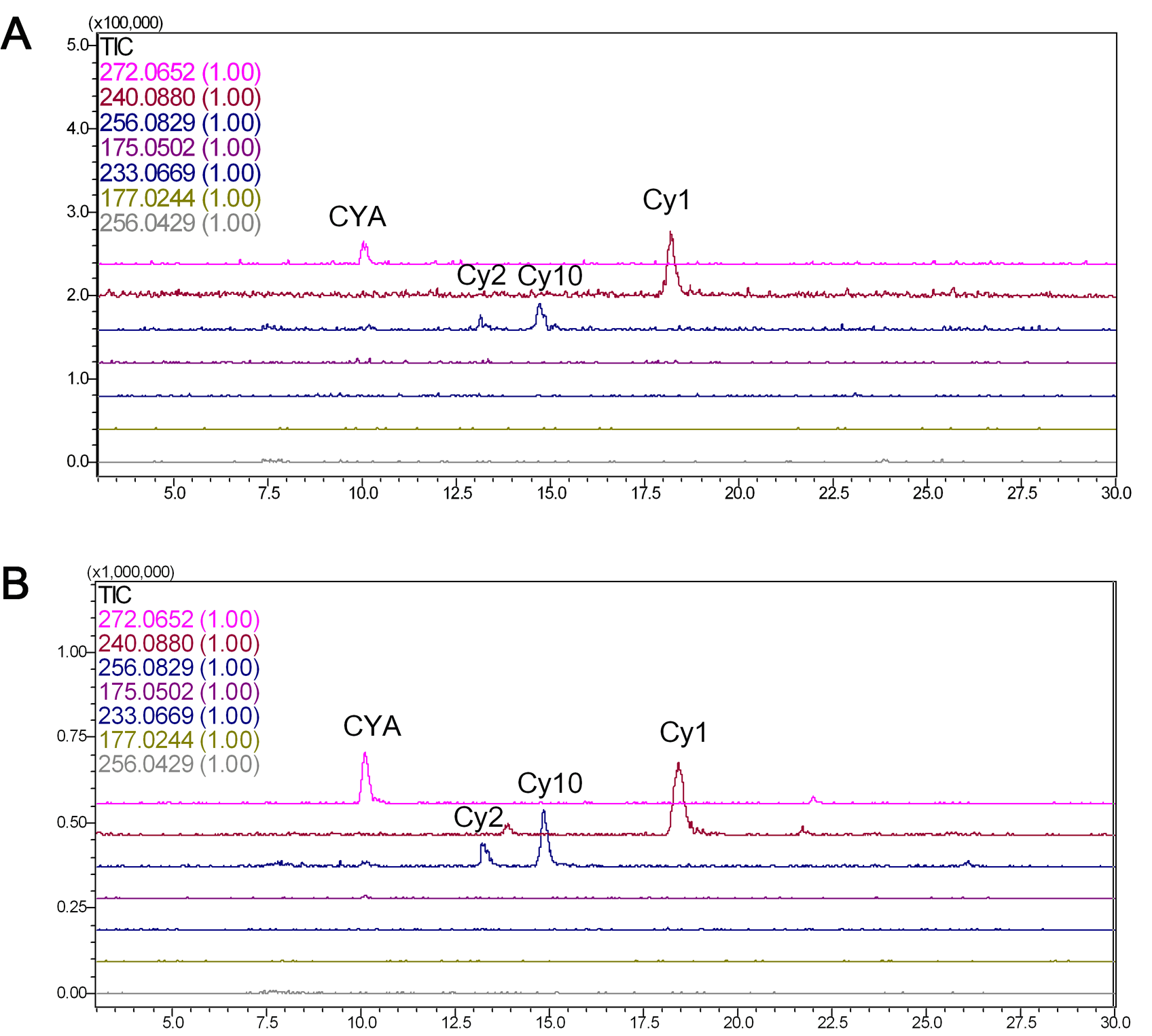

Supplement: S1 Fig — E. coli CVCC2943 cells were incubated with 4 μg/ml CYA under aerobic (A) and anaerobic conditions (B) for 0.5 h. The extracts of the metabolites from the bacteria were subjected to HPLC/ESI-IT-TOF MS as described in materials and methods. (TIF) [file pone.0136450.s001.tif]

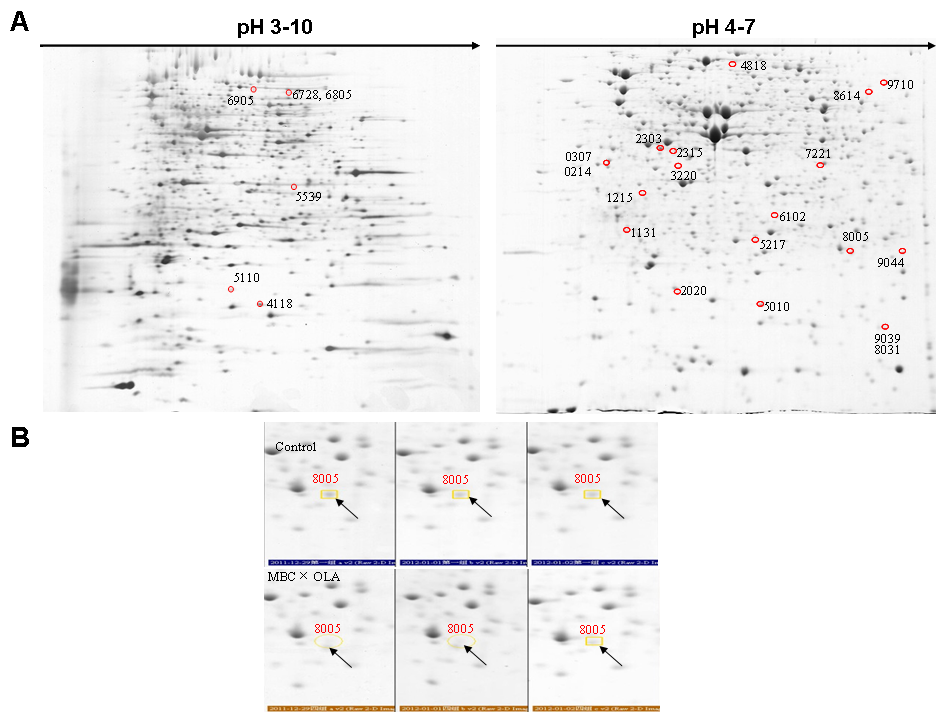

Supplement: S2 Fig — (A) Distribution of the differentially expressed proteins of E. coli CVCC2943 treated with CYA and OLA in the pH 3–10 and pH 4–7 2-D gels. (B) The differentially expressed protein No. 8005 (EftU1) was present in three replicated 2-D gels from the control group and the group treated with the MBC of OLA. Protein No. 8005 was down-regulated with 99% statistical significance. (TIF) [file pone.0136450.s002.tif]

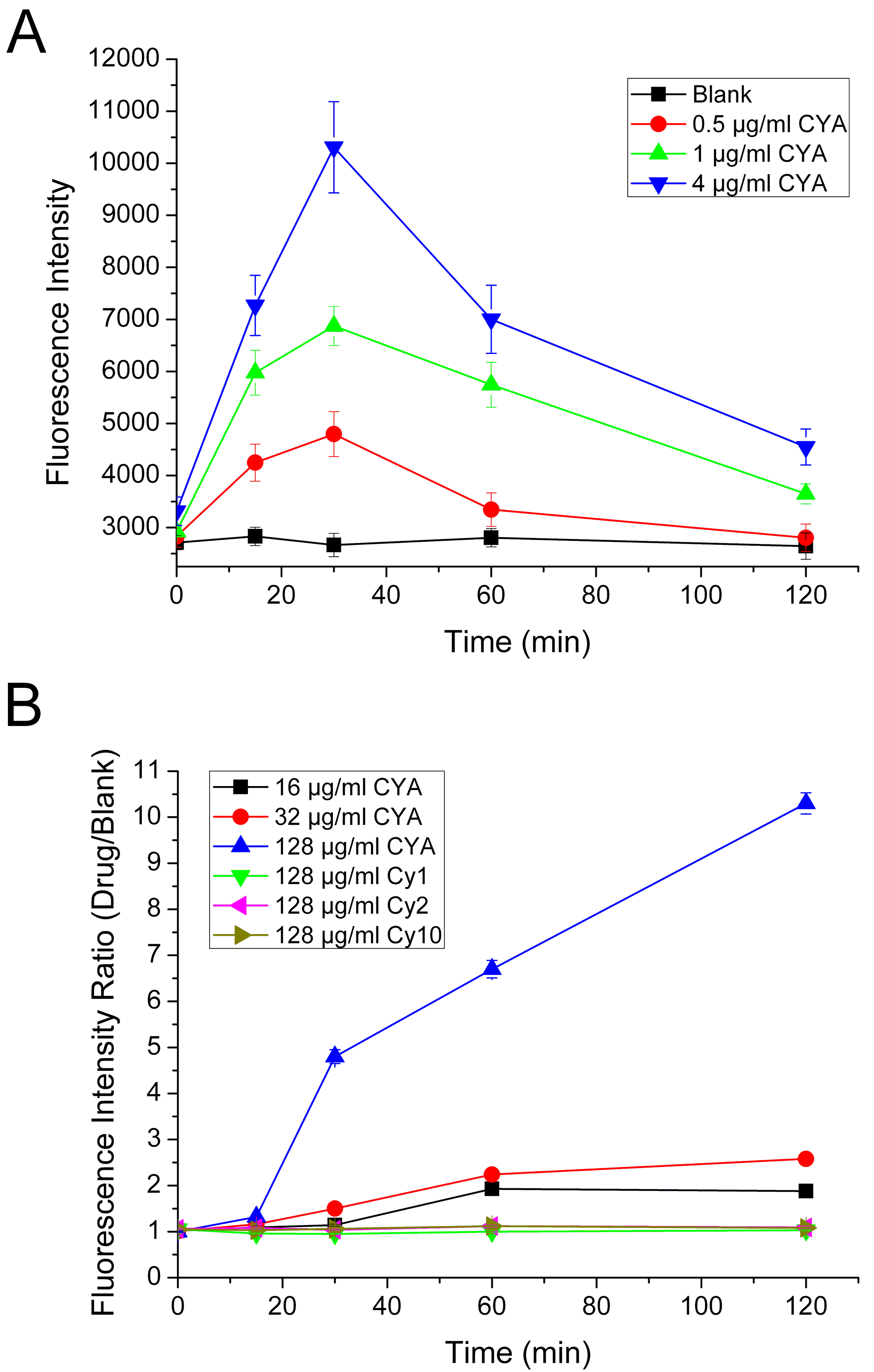

Supplement: S3 Fig — (A) Under anaerobic condition, E. coli CVCC2943 cells were treated with the indicated concentration of CYA, and 0.3% DMSO was used as a blank. After incubation for the indicated times, the level of ROS was detected as described in materials and methods. (B) Under aerobic conditions, the bacteria were treated with the indicated concentration of CYA, and 10% DMSO was used as a blank. After incubation of the bacteria with drugs for the indicated times, the superoxide radial levels were detected as described in materials and methods. The fluorescence intensity ratio was calculated as the fluorescence intensity of the drug-treated sample to the fluorescence intensity of the blank sample. The data were presented as the means ± SDs (error bars), n = 3. (TIF) [file pone.0136450.s003.tif]

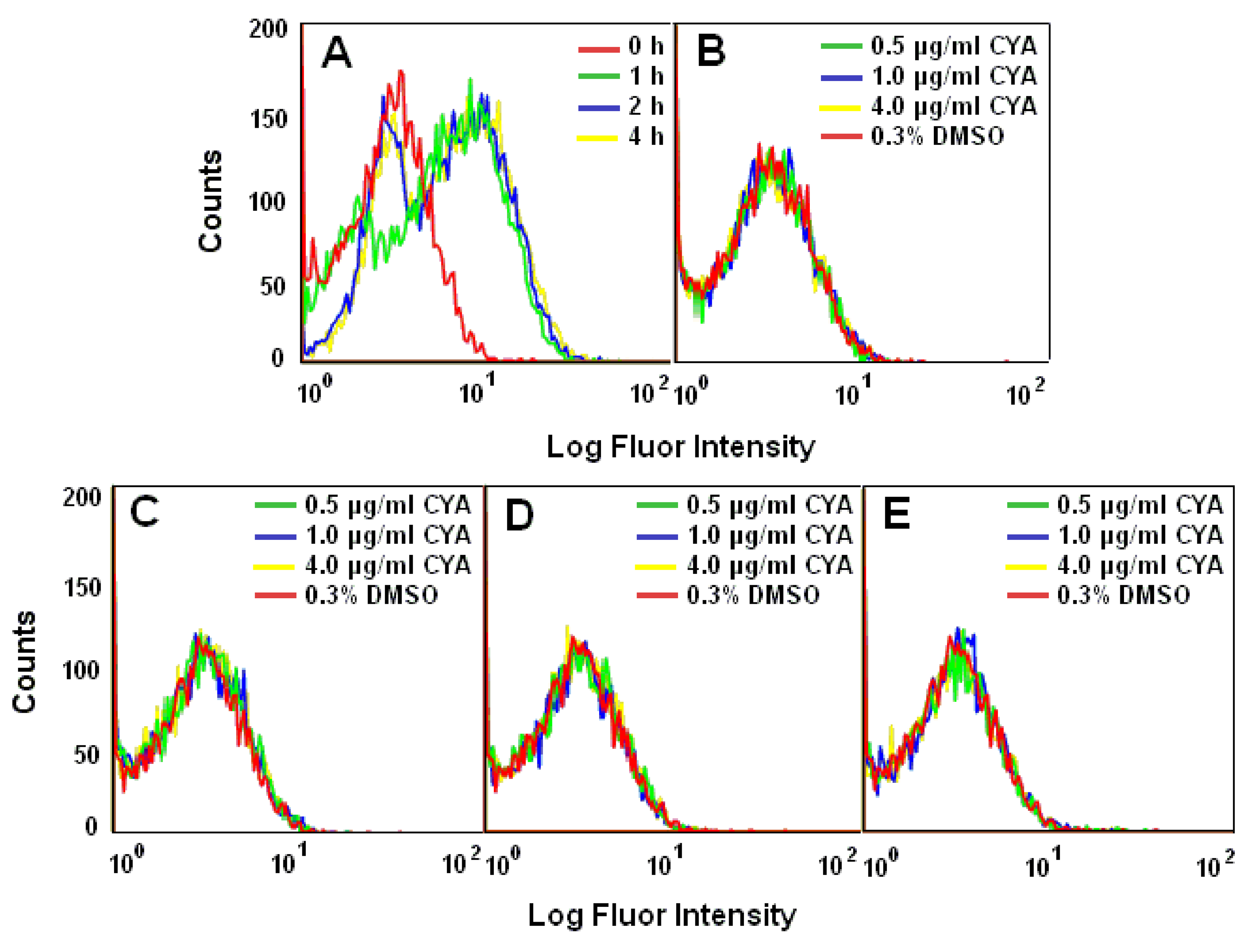

Supplement: S4 Fig — Bacteria were treated with 0.5, 1, and 4 μg/ml CYA or with 0.3% DMSO as a negative control for 0.5 h (B), 1 h (C), 2 h (D) and 4 h (E) under anaerobic conditions. The bacteria were exposed to 5 μg/ml carbenicillin as a positive control for 0, 1, 2 and 4 h, respectively (A). The levels of ·OH radicals were detected as described in materials and methods. (TIF) [file pone.0136450.s004.tif]

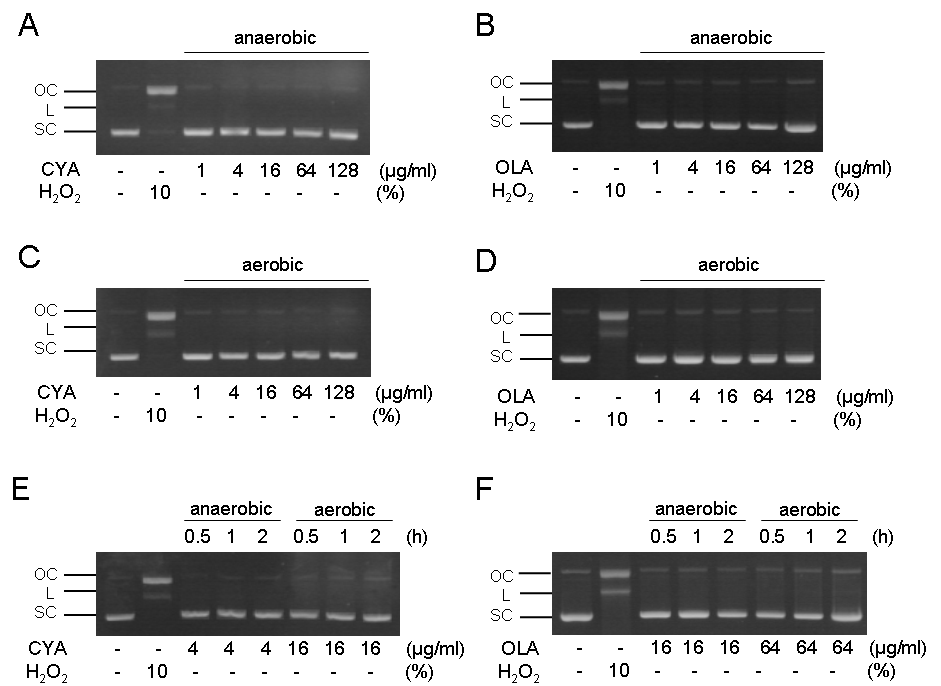

Supplement: S5 Fig — Supercoiled pBR322 DNA (10 μg/ml) was incubated with the indicated concentration of CYA (A, C, and E) or OLA (B, D, and F) at 37°C for 0.5 h (A-D) or the indicated times (E, F) under anaerobic or aerobic conditions. The treated plasmids were electrophoretically separated as described in materials and methods. H2O2 was set as a positive control. SC, L and OC indicate supercoiled, linear and open circular DNA, respectively. (TIF) [file pone.0136450.s005.tif]

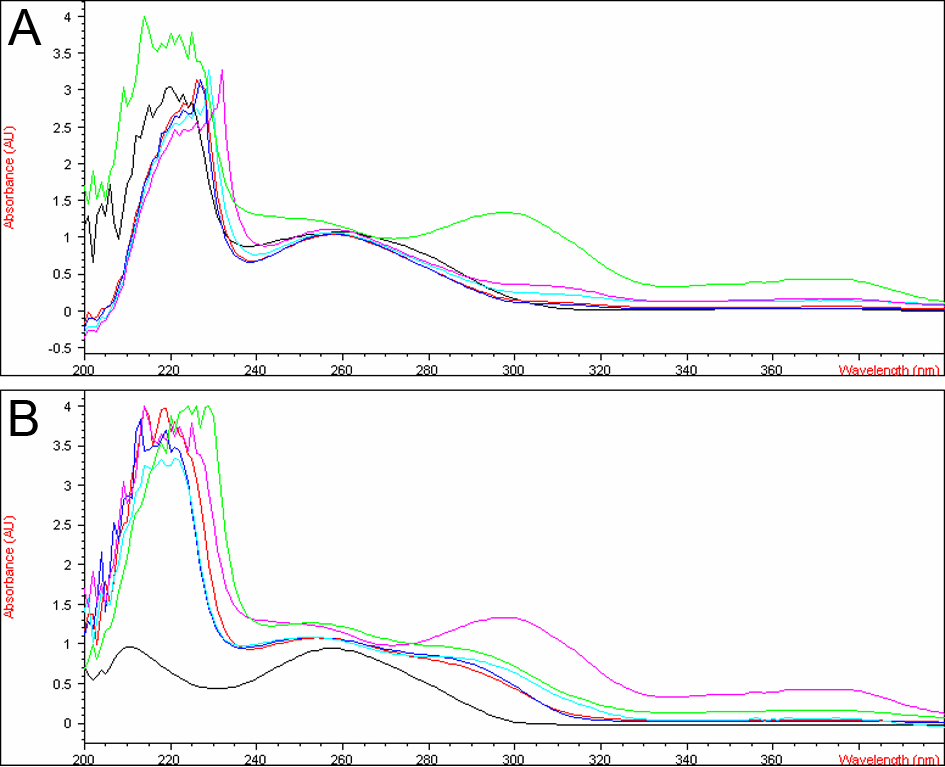

Supplement: S6 Fig — (A) 50 μg/ml DNA was incubated with CYA at a concentration of 0 μg/ml (black, the maximum UV absorption wavelength was located at 260 nm), 0.25 μg/ml (red, 258 nm), 0.5 μg/ml (blue, 258 nm), 1 μg/ml (cyan, 258 nm), and 2 μg/ml (magenta, 258 nm). The spectrum of 2 μg/ml CYA is indicated by a green line (298 nm). (B) 50 μg/ml DNA was incubated with CYA at a concentration of 0 μg/ml (black, 259 nm), 0.25 μg/ml (red, 254 nm), 0.5 μg/ml (blue, 253 nm), 1 μg/ml (cyan, 252 nm), and 2 μg/ml (green, 251 nm) in the presence of XO/X. The spectrum of CYA is indicated by a magenta line (298 nm). (TIF) [file pone.0136450.s006.tif]
