# Supplementary material for: Systematic and Molecular Basis of the Antibacterial Action of Quinoxaline 1,4-Di-N-Oxides against Escherichia coli
Source: PLoS One. 2015 Aug 21;10(8):e0136450. doi: 10.1371/journal.pone.0136450 (PMC4546592; doi:10.1371/journal.pone.0136450)
Supplement: S1 Table — (DOC) [file pone.0136450.s007.doc]

**S1 Table. Differentially expressed genes of *E. coli* CVCC2943** in response to cyadox and olaquindox.

| **Gene description/Protein encoded** | **Gene** | **Fold-Change** | | | | |
| --- | --- | --- | --- | --- | --- | --- |
|  |  | **0.5**×**MIC CYA** | | **MBC CYA** | **MIC OLA** | **MBC OLA** |
| **1. SOS response** | | | | | | |
| DNA strand exchange and recombination protein with protease and nuclease activity | *recA* | **7.46^a^** | | **11.42** | **9.70** | **13.88** |
| LexA DNA-binding transcriptional repressor | *lexA* | **4.07** | | **4.54** | **4.30** | **4.68** |
| SOS cell division inhibitor | *sulA* | **8.59** | | **17.59** | **13.50** | **15.36** |
| Recombination and repair protein | *recN* | **9.95** | | **21.52** | **15.91** | **22.24** |
| Branch migration of Holliday structures; repair | *ruvA* | **2.39** | | **2.43** | **2.30** | **2.43** |
| DNA damage-inducible protein I | *dinI* | **3.86** | | **5.01** | **4.57** | **5.47** |
| Recombination regulator RecX / regulatory protein for RecA | *recX* | **3.33** | | **6.11** | **5.10** | **6.05** |
| DNA repair; excision nuclease subunit B | *uvrB* | **3.18** | | **5.15** | **3.05** | **6.75** |
| Excinuclease ABC subunit A | *uvrA* | **2.78** | | **3.75** | **4.20** | **4.14** |
| Nucleotide excision repair endonuclease | *ydjQ* | **2.41** | | **2.64** | **2.98** | **2.22** |
| DNA gyrase inhibitor | *sbmC* | **2.24** | | **2.74** | **4.27** | **2.91** |
| DNA-damage-inducible protein D | *dinD* | **2.72** | | **4.43** | **5.48** | **5.24** |
| DNA-damage-inducible SOS response protein | *dinF* | **2.48** | | **2.44** | **2.58** | **2.49** |
| DNA damage-inducible protein YebG | *yebG* | **9.26** | | **18.56** | **12.35** | **22.28** |
| DNA polymerase II | *polB* | **2.07** | | **2.45** | **2.79** | **2.96** |
| SOS mutagenesis; error-prone repair; processed to UmuD'; forms complex with UmuC(pol V) | *umuD* | 1.90^b^ | | **4.78** | **2.97** | **4.10** |
| SOS mutagenesis and repair(pol V) | *umuC* | 1.77 | | **4.50** | **2.70** | **4.09** |
| Branch migration of Holliday structures; repair helicase | *ruvB* | 1.98 | | **2.14** | **2.08** | 1.95 |
| DNA polymerase IV | *dinB* | 1.48 | | 1.32 | **2.01** | 1.78 |
| **2. Cell envelope** | | | | | | |
| Lipoprotein component of BamABCDE OM biogenesis complex | *smpA* | | 1.77 | **2.52** | **2.21** | **2.94** |
| Inner membrane transport protein YdhC | *ydhC* | | 1.61 | **2.89** | 1.52 | **2.10** |
| Glycine betaine transporter ATP-binding subunit | *proV* | | 1.05 | **-2.04** | 1.06 | **-2.15** |
| Ribose ABC transporter permease protein | *rbsC* | | -1.14 | **-2.41** | -1.40 | **-2.86** |
| PTS system trehalose(maltose)-specific transporter subunits IIBC | *treB* | | -1.06 | **-2.80** | -1.54 | **-2.82** |
| D-galactonate transporter | *dgoT* | | -1.17 | -1.13 | **-2.18** | -1.07 |
| Confers peroxide resistance in biofilms | *bsmA* | | 1.15 | -1.16 | **2.64** | -1.27 |
| Transmembrane signal transducer for ferric citrate transporter | *fecR* | | -1.14 | 1.01 | **2.07** | 1.14 |
| Transporter protein | *pgtP* | | -1.25 | -1.26 | **-2.15** | -1.41 |
| Regulatory protein | *pgtC* | | -1.14 | -1.56 | **-2.30** | -1.69 |
| Regulatory protein | *pgtB* | | -1.04 | -1.37 | **-2.31** | -1.68 |
| Small toxic polypeptide- | *hokB* | | -1.34 | -1.64 | **-2.97** | -1.73 |
| Glycerol facilitator protein | *glpF* | | -1.09 | -1.50 | **2.71** | -1.71 |
| PTS system, galactitol-specific IIC component | *gatC* | | -1.21 | -1.39 | **-2.05** | -1.61 |
| Outer-membrane lipoprotein carrier protein | *lolA* | | 1.55 | 1.84 | **2.02** | 1.88 |
| Multidrug resistance protein Y | *emrY* | | -1.08 | -1.50 | **-2.41** | -1.13 |
| Biofilm, cell surface and signaling protein | *bhsA* | | 1.10 | 1.97 | 1.67 | **2.43** |
| Glycine betaine transporter membrane protein | *proW* | | -1.27 | -1.70 | -1.21 | **-2.17** |
| Maltose/maltodextrin transporter ATP-binding protein | *malK* | | -1.14 | -1.48 | -1.22 | **-2.12** |
| **3. Virulence genes** | | | | | | |
| Integrase for prophage | *intT* | | **2.32** | **5.31** | **4.66** | **6.27** |
| Toxin YafO | *yafO* | | 1.96 | **2.26** | **2.10** | **2.51** |
| Flagellar motor switch protein FliM | *fliM* | | -1.12 | **-2.21** | -1.89 | **-2.07** |
| Periplasmic repressor CpxP / inhibitor of the cpx response; periplasmic adaptor | *cpxP* | | 1.31 | 1.73 | **2.98** | **2.06** |
| PapD protein | *papD_2* | | -1.35 | -1.53 | **-2.04** | **-2.00** |
| Flagellar basal body-associated protein FliL | *fliL* | | -1.04 | -1.92 | **-2.02** | -1.87 |
| Flagellar basal-body MS-ring and collar protein | *fliF* | | -1.39 | -1.66 | **-2.00** | -1.71 |
| Tail fiber assembly protein | *ydfM* | | -1.17 | -1.11 | **-2.05** | -1.63 |
| PapD protein | *papD* | | -1.40 | -1.42 | **-2.13** | -1.66 |
| PapF protein | *papF* | | -1.10 | -1.23 | **-2.04** | -1.44 |
| PapG protein | *papG* | | -1.21 | -1.23 | **-2.08** | -1.45 |
| PapI protein | *papI* | | -1.07 | -1.38 | **-3.18** | -1.70 |
| Modulator of drug activity B | *mdaB* | | 1.59 | 1.79 | 1.23 | **2.81** |
| DNA starvation/stationary phase protection protein Dps | *dps* | | 1.25 | 1.38 | 1.74 | **2.13** |
| Putative antitoxin of the YafO-YafN toxin-antitoxin system | *yafN* | | 1.92 | 1.99 | 1.84 | **2.31** |
| Prophage protein | *ninE* | | -1.08 | -1.22 | -1.70 | **2.44** |
| Acid-resistance protein | *hdeA* | | 1.23 | 1.25 | 1.35 | **2.10** |
| Superoxide dismutase | *sodB* | | 1.35 | 1.37 | 1.57 | **2.24** |
| **4. Metabolism of carbohydrates, amino acids and purine/pyrimidine** | | | | | | |
| Glycosyl transferase family protein | *ybiB* | | 1.87 | **2.79** | **2.54** | **2.45** |
| Ribonucleotide-diphosphate reductase subunit alpha | *nrdA* | | 1.36 | **2.32** | **2.11** | **2.54** |
| Ribonucleotide-diphosphate reductase subunit beta | *nrdB* | | 1.53 | **3.10** | **3.29** | **3.61** |
| Ferredoxin-type protein | *napF* | | -1.78 | **-2.12** | **-3.22** | **-3.43** |
| 2Fe-2S ferredoxin YfaE | *yfaE* | | 1.25 | **2.41** | **2.54** | **2.84** |
| Lysine decarboxylase, inducible | *cadA* | | -1.02 | **-2.17** | **-2.70** | 1.17 |
| Intracellular pH elevation | *cadB* | | -1.19 | **-2.04** | **-3.36** | -1.01 |
| Hydrogenase-1 operon protein HyaE | *hyaE* | | -1.76 | **-2.15** | -1.77 | **-2.43** |
| Trehalose-6-phosphate hydrolase | *treC* | | -1.13 | **-2.56** | -1.59 | **-2.55** |
| Activation of the ribonucleoside triphosphate reductase | *nrdG* | | 1.15 | **2.02** | 1.19 | 1.90 |
| Assembly protein for periplasmic nitrate reductase | *napD* | | -1.87 | -1.81 | **-2.80** | **-3.05** |
| Phosphoadenosine phosphosulfate reductase | *cysH* | | 1.51 | 1.72 | **2.26** | **2.11** |
| Translation inhibitor protein RaiA | *yfiA* | | 1.23 | 1.38 | **6.26** | **3.02** |
| Ketol-acid reductoisomerase | *ilvC* | | 1.02 | 1.02 | **2.49** | 1.11 |
| Glucosamine-6-phosphate deaminase | *nagB* | | 1.32 | -1.04 | **2.50** | 1.39 |
| Ethanolamine utilization protein eutE | *c4534* | | -1.21 | -1.33 | **-2.14** | -1.46 |
| Ethanolamine utilization protein eutJ | *c4526* | | -1.23 | -1.42 | **-2.01** | -1.57 |
| Glycerol-3-phosphate dehydrogenase | *glpD* | | 1.12 | 1.67 | **2.98** | 1.12 |
| D-ribose pyranase | *rbsD* | | 1.33 | -1.47 | **2.12** | -1.09 |
| Aspartate/ornithine carbamoyltransferase family protein | *ygeW* | | -1.10 | -1.16 | **-2.38** | -1.34 |
| Formate dehydrogenase-O, major subunit | *fdoG* | | 1.08 | -1.13 | **2.25** | -1.26 |
| Inositol monophosphatase | *suhB* | | 1.05 | 1.21 | **-2.96** | -1.20 |
| N-acetylneuraminic acid mutarotase | *nanM* | | -1.16 | -1.22 | **-2.02** | -1.47 |
| Pyridine nucleotide-disulfide oxidoreductase | *ykgC* | | -1.19 | -1.23 | **-2.25** | -1.51 |
| IlvB operon leader peptide | *ivbL* | | 1.16 | -1.41 | **2.47** | -1.40 |
| Ais protein /protein induced by aluminum | *ais* | | 1.13 | -1.31 | **-2.97** | -1.40 |
| 50S ribosomal protein L31 type B | *rpmE2* | | -1.25 | -1.55 | **-2.45** | -1.61 |
| 30S ribosomal protein S15 | *rpsO* | | 1.55 | 1.53 | 1.99 | **2.20** |
| Serine hydroxymethyltransferase | *glyA* | | 1.65 | 1.66 | 1.43 | **3.50** |
| Periplasmic protein disulfide isomerase I | *dsbA* | | 1.28 | 1.74 | 1.60 | **2.25** |
| Serine/threonine protein kinase | *rdoA* | | 1.32 | 1.63 | 1.46 | **2.27** |
| D-alanyl-D-alanine carboxypeptidase fraction A | *dacA* | | 1.33 | 1.48 | 1.06 | **2.02** |
| D-serine dehydratase | *dsdA* | | 1.13 | -1.20 | -1.38 | **2.37** |
| Guanosine 5'-monophosphate oxidoreductase | *guaC* | | 1.45 | 1.75 | 1.43 | **2.27** |
| Anaerobic ribonucleoside triphosphate reductase | *nrdD* | | 1.12 | 1.82 | 1.16 | **2.16** |
| Maltose ABC transporter periplasmic protein | *malE* | | -1.26 | -1.76 | -1.43 | **-2.49** |
| [Citrate [pro-3S]-lyase] ligase | *citC* | | 1.06 | -1.41 | -1.94 | **-2.12** |
| 2-hydroxyglutaryl-CoA dehydratase | *yjiM* | | -1.23 | -1.32 | -1.32 | **-2.10** |
| 50S ribosomal protein L32 | *rpmF* | | 1.40 | 1.67 | 1.46 | **2.11** |
| ATP-dependent protease peptidase subunit | *hslV* | | 1.34 | 1.76 | 1.41 | **2.49** |
| Phosphodiesterase | *yfcE* | | 1.24 | 1.54 | 1.15 | **2.10** |
| **5. Cytochrome** | | | | | | |
| Succinate dehydrogenase cytochrome b556 large membrane subunit | *sdhC* | | 1.29 | 1.07 | **5.80** | 1.14 |
| Succinate dehydrogenase cytochrome b556 small membrane subunit | *sdhD* | | -1.07 | -1.14 | **3.41** | -1.22 |
| Cytochrome o ubiquinol oxidase subunit II | *cyoA* | | 1.44 | 1.27 | **3.76** | 1.28 |
| Cytochrome o ubiquinol oxidase subunit III | *cyoC* | | 1.04 | -1.15 | **3.33** | -1.25 |
| Cytochrome o ubiquinol oxidase subunit I | *cyoB* | | 1.04 | -1.08 | **2.65** | 1.04 |
| **6. DNA-binding transcriptional regulator** | | | | | | |
| DNA-binding transcriptional activator MarA | *marA* | | 1.30 | **2.02** | 1.01 | **3.01** |
| DNA-binding transcriptional regulator EnvR | *envR* | | -1.31 | -1.29 | **-2.14** | -1.41 |
| DNA-binding transcriptional regulator MelR | *melR* | | 1.07 | -1.41 | **4.48** | -1.07 |
| LysR family transcriptional regulator | *c0411* | | -1.04 | -1.53 | **-2.03** | -1.65 |
| Transcriptional regulator BetI | *betI* | | 1.02 | 1.04 | **2.04** | -1.01 |
| DNA-binding transcriptional repressor MarR | *marR* | | 1.08 | 1.49 | -1.22 | **2.12** |
| **7. Unknown function** | | | | | | |
| Hypothetical protein /inner membrane protein regulate | *ydjM* | | **2.33** | **2.46** | **2.56** | **2.31** |
| Hypothetical protein /secreted protein | *yebF* | | **7.22** | **20.03** | **9.17** | **15.67** |
| DNA-damage-inducible protein I / hypothetical protein | *c3144* | | **2.52** | **8.71** | **6.82** | **11.16** |
| Putative lipase | *c4836* | | **2.28** | **2.31** | **3.73** | **2.55** |
| Hypothetical protein | *c0943* | | **12.13** | **44.19** | **31.84** | **62.06** |
| Hypothetical protein | *c1094* | | **2.01** | **3.99** | **3.62** | **3.85** |
| Putative stress-response protein | *yjbJ* | | **2.47** | **3.11** | 1.69 | **2.88** |
| Hypothetical protein /DNA recombination protein RmuC | *rmuC* | | **2.06** | 1.68 | **2.56** | **2.31** |
| Hypothetical protein / inner membrane protein, UPF029 | *yohJ* | | 1.24 | **2.02** | 1.33 | **2.37** |
| Hypothetical protein / inner membrane protein, LrgB | *yohK* | | 1.25 | **2.01** | 1.57 | **2.29** |
| Putative cI repressor protein | *c1546* | | 1.50 | **3.03** | 1.93 | **3.63** |
| Putative chaperone | *yegD* | | 1.28 | **2.30** | 1.34 | **2.28** |
| Hypothetical protein | *c1545* | | 1.82 | **2.78** | 1.99 | **4.03** |
| Hypothetical protein | *c1524* | | -1.36 | **2.84** | 1.26 | 1.38 |
| Hypothetical protein | *c0583* | | 1.33 | **2.42** | -1.06 | 1.36 |
| Hypothetical protein | *c2318* | | 1.27 | **2.07** | -1.40 | 1.68 |
| Hypothetical protein | *c0859* | | 1.43 | **2.02** | 1.41 | 1.86 |
| Hypothetical protein / predicted inner membrane prote | *ygaP* | | 1.15 | 1.42 | **3.48** | **2.08** |
| CP4-like integrase | *c0391* | | -1.13 | -1.68 | **-3.19** | **-2.20** |
| Hypothetical protein | *Z3347* | | 1.34 | 1.05 | **-2.21** | **4.82** |
| Hypothetical protein | *c0693* | | 1.41 | 1.56 | **2.30** | **2.03** |
| Hypothetical protein | *c5205* | | -1.05 | -1.51 | **-3.30** | **-2.23** |
| Hypothetical protein | *c5175* | | -1.20 | -1.56 | **-3.25** | **-2.20** |
| Hypothetical protein | *c0398* | | -1.23 | -1.47 | **-2.29** | **-2.07** |
| Hypothetical protein | *c4556* | | -1.27 | -1.35 | **-2.72** | **-2.15** |
| Hypothetical protein | *c5154* | | -1.35 | -1.40 | **-2.33** | **-2.05** |
| Hypothetical protein | *Z1663* | | -1.09 | -1.60 | **-3.46** | **-2.15** |
| Hypothetical protein | *c2484* | | -1.37 | -1.60 | **-2.77** | **-2.64** |
| Putative aldo/keto reductase | *c0413* | | -1.06 | -1.47 | **-2.33** | -1.50 |
| Oxidoreductase ydfI | *ydfI* | | -1.23 | -1.19 | **-2.06** | -1.26 |
| Hypothetical protein / putative transcriptional regulator | *c5206* | | -1.25 | -1.60 | **-2.72** | -1.99 |
| Insertion element IS1 1/2/3/5/6 protein insA | *c0396* | | -1.17 | -1.45 | **-2.08** | -1.75 |
| Transposase | *c5176* | | -1.11 | -1.54 | **-3.32** | -1.68 |
| InsB protein | *c0397* | | 1.17 | 1.17 | **-2.46** | -1.22 |
| Putative NADH-dependent flavin oxidoreductase | *c0409* | | 1.02 | -1.57 | **-2.34** | -1.53 |
| Putative oxidoreductase | *yeiQ* | | 1.06 | -1.00 | **2.67** | -1.06 |
| Putative minor fimbrial subunit precursor | *c4210* | | -1.07 | -1.12 | **-2.03** | -1.14 |
| Predicted 4Fe-4S ferridoxin-type | *ydhY* | | 1.01 | -1.47 | **-2.01** | -1.39 |
| Hypothetical protein / lipoprotein, function unknown | *yecR* | | -1.10 | -1.31 | **-2.25** | -1.53 |
| Putative oxidoreductase | *yqfA* | | 1.23 | 1.31 | **2.52** | 1.63 |
| Putative oxidoreductase | *ydeP* | | -1.04 | -1.39 | **-2.05** | -1.83 |
| Transcriptional regulator YdeO | *ydeO* | | -1.13 | -1.42 | **-2.42** | -1.71 |
| Hypothetical protein | *yegR* | | -1.20 | -1.13 | **-2.13** | -1.24 |
| Hypothetical protein | *ydcH* | | 1.10 | 1.05 | **2.04** | 1.03 |
| Hypothetical protein | *matC* | | -1.17 | -1.38 | **-2.04** | -1.81 |
| Hypothetical protein | *ykgL* | | -1.14 | -1.39 | **-2.04** | -1.60 |
| Hypothetical protein | *yfdX* | | -1.10 | -1.34 | **-2.11** | -1.57 |
| Hypothetical protein | *c4557* | | -1.09 | -1.14 | **-2.21** | -1.68 |
| Hypothetical protein | *c0392* | | -1.08 | -1.50 | **-3.50** | -1.76 |
| Hypothetical protein | *c0174* | | -1.27 | -1.30 | **-2.24** | -1.54 |
| Hypothetical protein | *c3677* | | 1.04 | -1.10 | **-2.13** | -1.32 |
| Hypothetical protein | *c1008* | | -1.25 | -1.27 | **-2.02** | -1.84 |
| Hypothetical protein | *c3919* | | 1.48 | 1.68 | **2.26** | 1.75 |
| Hypothetical protein | *c5195* | | -1.08 | -1.26 | **-2.14** | -1.83 |
| Hypothetical protein | *c5190* | | -1.17 | -1.56 | **-3.79** | -1.75 |
| Hypothetical protein | *c2230* | | 1.26 | 1.06 | **2.05** | 1.65 |
| Hypothetical protein | *c4081* | | 1.22 | 1.03 | **2.00** | 1.16 |
| Hypothetical protein | *c5199* | | -1.09 | -1.39 | **-3.87** | -1.59 |
| Hypothetical protein | *c1809* | | -1.24 | -1.63 | **-2.11** | -1.92 |
| Hypothetical protein | *c3684* | | -1.10 | -1.35 | **-2.36** | -1.69 |
| Hypothetical protein | *c5209* | | -1.15 | -1.39 | **-2.39** | -1.60 |
| Hypothetical protein | *c1915* | | -1.29 | -1.67 | **-2.37** | -1.62 |
| Hypothetical protein | *c5172* | | -1.12 | -1.29 | **-2.30** | -1.72 |
| Hypothetical protein | *c0813* | | 1.53 | 1.75 | **2.08** | 1.69 |
| Hypothetical protein | *c4311* | | 1.05 | 1.19 | **-2.001** | -1.06 |
| Hypothetical protein | *c1956* | | -1.21 | -1.43 | **-2.04** | -1.39 |
| Hypothetical protein | *c4579* | | -1.23 | -1.58 | **-2.44** | -1.77 |
| Hypothetical protein | *c4090* | | 1.12 | 1.38 | **2.46** | 1.47 |
| Hypothetical protein | *c5212* | | -1.15 | -1.57 | **-2.39** | -1.65 |
| Hypothetical protein | *c5161* | | -1.13 | -1.98 | **-2.82** | -1.28 |
| Hypothetical protein | *c5169* | | -1.25 | -1.49 | **-2.93** | -1.87 |
| Hypothetical protein | *c5171* | | -1.15 | -1.46 | **-2.81** | -1.87 |
| Hypothetical protein | *c2481* | | -1.11 | -1.26 | **-2.13** | -1.70 |
| Hypothetical protein | *c4938* | | 1.15 | 1.43 | **2.59** | 1.73 |
| Hypothetical protein | *c2757* | | 1.33 | 1.54 | **2.34** | 1.42 |
| Hypothetical protein | *c5173* | | -1.01 | -1.32 | **-2.18** | -1.41 |
| Hypothetical protein | *c0394* | | 1.05 | -1.45 | **-2.98** | -1.59 |
| Hypothetical protein | *c5170* | | -1.08 | -1.52 | **-3.85** | -1.94 |
| Hypothetical protein | *c5200* | | 1.05 | -1.74 | **-2.91** | -1.23 |
| Hypothetical protein | *c5211* | | 1.08 | -1.17 | **-2.23** | -1.40 |
| Hypothetical protein | *ECs5267* | | -1.04 | 1.19 | **2.14** | -1.13 |
| Hypothetical protein | *c4078* | | 1.40 | 1.28 | **2.02** | 1.82 |
| Hypothetical protein | *c1929* | | -1.06 | -1.49 | **-2.98** | -1.83 |
| Hypothetical protein | *c4084* | | 1.21 | 1.17 | **2.10** | 1.31 |
| Hypothetical protein | *c2114* | | 1.19 | 1.20 | **2.05** | 1.47 |
| Hypothetical protein | *c5210* | | -1.13 | -1.29 | **-2.44** | -1.89 |
| Hypothetical protein | *c0375* | | -1.30 | -1.54 | **-2.48** | -1.95 |
| Hypothetical protein | *c3336* | | -1.15 | -1.23 | **-2.45** | -1.51 |
| Hypothetical protein | *c3487* | | -1.505 | -1.63 | **-2.54** | -1.80 |
| Hypothetical protein | *c2903* | | -1.01 | -1.41 | **-2.63** | 1.48 |
| Hypothetical protein | *c5155* | | 1.04 | -1.37 | **-2.25** | -1.40 |
| unknown /ncRNA | *micF* | | 1.50 | 1.74 | 1.03 | **2.44** |
| ncRNA | *glmZ* | | 1.50 | 1.60 | 1.57 | **2.09** |
| hypothetical protein / conserved protein, UPF0076 fam | *yoaB* | | 1.54 | 1.70 | 1.58 | **2.25** |
| hypothetical protein | *yqjD* | | 1.26 | 1.40 | 1.34 | **2.01** |
| hypothetical protein | *yiaF* | | 1.37 | 1.48 | 1.55 | **2.01** |
| putative cI repressor protein | *c1546* | | 1.41 | 1.82 | 1.83 | **2.62** |
| putative cII antiterminator protein for prophage CP-933 | *Z0310* | | -1.17 | -1.07 | -1.85 | **6.26** |
| hypothetical protein | *c1544* | | 1.39 | 1.94 | 1.86 | **2.50** |
| hypothetical protein | *c0258* | | -1.37 | -1.35 | -1.52 | **2.28** |
| hypothetical protein | *Z1441* | | -1.06 | 1.01 | -1.83 | **14.12** |
| hypothetical protein | *Z1428* | | -1.09 | 1.33 | -1.64 | **9.25** |

0.5MIC , MIC, MBC×, inhibitor concentrations.

^a^ Numbers in bold indicate the value of the fold change for the genes that are significantly up-expressed or down-expressed，at least the gene in one of the four comparative group is ≥2 or ≤-2.

^b^ Numbers in normal represent the fold change value >-2 and <2.
