# Supplementary material for: Systematic and Molecular Basis of the Antibacterial Action of Quinoxaline 1,4-Di-N-Oxides against Escherichia coli
Source: PLoS One. 2015 Aug 21;10(8):e0136450. doi: 10.1371/journal.pone.0136450 (PMC4546592; doi:10.1371/journal.pone.0136450)
Supplement: S2 Table — (DOC) [file pone.0136450.s008.doc]

**S2 Table. Differentially expressed proteins of *E. coli* CVCC2943 in response to CYA from the pH 3-10 2-D gel.**

| Group | Spot name | Protein name | Change | Mass | PI | Accession number | Protein description | Mascot score |
| --- | --- | --- | --- | --- | --- | --- | --- | --- |
| 0.5×MIC CYA | 4118 | Dps | >2 | 18683.7 | 5.72 | B7UM08 | DNA protection during starvation protein | 172 |
|  | 5539 | FolD | Appear | 31221.4 | 5.91 | B7UKK6 | Bifunctional protein | 121 |
|  | 6805 | NuoC/D | Appear | 68450.6 | 5.98 | Q0TFG0 | NADH-quinone oxidoreductase subunit C/D | 189 |
| MBC CYA | 6728 | NuoC/D | Appear | 68450.6 | 5.98 | Q0TFG0 | NADH-quinone oxidoreductase subunit C/D | 197 |
|  | 5110 | Ssb | Appear | 18963.3 | 5.44 | P0AGE1 | Single-stranded DNA-binding protein | 127 |
|  | 6905 | NarY | Appear | 59546 | 5.66 | P19318 | Respiratory nitrate reductase 2 beta chain | 96 |
