# Supplementary material for: Systematic and Molecular Basis of the Antibacterial Action of Quinoxaline 1,4-Di-N-Oxides against Escherichia coli
Source: PLoS One. 2015 Aug 21;10(8):e0136450. doi: 10.1371/journal.pone.0136450 (PMC4546592; doi:10.1371/journal.pone.0136450)
Supplement: S3 Table — (DOC) [file pone.0136450.s009.doc]

**S3 Table. Differentially expressed proteins of *E. coli* CVCC2943 in response to CYA and OLA from the pH 4-7 2-D gel.**

| Group | Spot name | Protein name | Change | Mass | PI | Accession number | Protein description | Mascot score |
| --- | --- | --- | --- | --- | --- | --- | --- | --- |
| 0.5×MIC CYA | 2315 | MreB | Missed | 37100 | 5.19 | P0A9X4 | Rod shape-determining protein, | 196 |
|  | 2020 | EftU1 | Missed | 43427.4 | 5.3 | A8A5E6 | Elongation factor Tu 1 | 60 |
|  | 8031 | YfbU | Missed | 19646.5 | 6.16 | B7UFV2 | UPF0304 protein yfbU | 135 |
|  | 0307 | CH60 | Missed | 57463.8 | 4.85 | A7ZV12 | 60 kDa chaperonin | 57 |
|  | 4818 | RpoC | Missed | 155918.3 | 6.67 | P0A8T8 | DNA-directed RNA polymerase subunit beta | 56 |
|  | 7221 | PflB | Missed | 85587.8 | 5.69 | P09373 | Formate acetyltransferase 1 | 58 |
| MBC CYA | 9044 | LexA | Missed | 22343.9 | 6.23 | B7UPK4 | LexA repressor | 211 |
| MIC OLA | 8005 | EftU1 | <-2 | 43427.4 | 5.3 | A8A5E6 | Elongation factor Tu 1 | 160 |
|  | 5217 | EftU1 | <-2 | 43427.4 | 5.3 | A8A5E6 | Elongation factor Tu 1 | 77 |
|  | 9044 | LexA | <-2 | 22343.9 | 6.23 | B7UPK4 | LexA repressor | 211 |
|  | 1215 | Odp1 | <-2 | 99948.1 | 5.46 | P0AFG8 | Pyruvate dehydrogenase E1 component | 74 |
|  | 2303 | IadA | <-2 | 41400.4 | 5.08 | P39377 | Isoaspartyl dipeptidase | 149 |
|  | 9039 | YfbU | Missed | 19646.5 | 6.16 | B7UFV2 | UPF0304 protein yfbU | 135 |
|  | 1131 | NanE | Missed | 24371.6 | 4.89 | B7UJV6 | Putative N-acetylmannosamine-6-phosphate 2-epimerase | 82 |
| MBC OLA | 5010 | Ssb | >2 | 18963.3 | 5.44 | P0AGE1 | Single-stranded DNA-binding protein, | 192 |
|  | 3220 | YgfZ | Appear | 36292.5 | 5.19 | Q0TDV3 | tRNA-modifying protein ygfZ | 74 |
|  | 0214 | CH60 | Missed | 57463.8 | 4.85 | A7ZV12 | 60 kDa chaperonin | 57 |
|  | 6102 | KpsU | <-2 | 27256 | 5.62 | P42216 | 3-deoxy-manno-octulosonate cytidylyltransferase | 85 |
|  | 8614 | FumA | <-2 | 60773.8 | 6.11 | P0AC34 | Fumarate hydratase class I, Fumarase | 149 |
|  | 9710 | SelB | <-2 | 68995.7 | 6.11 | P14081 | Selenocysteine-specific elongation factor | 144 |
|  | 9044 | LexA | <-2 | 22343.9 | 6.23 | B7UPK4 | LexA repressor | 211 |
