# Supplementary material for: Systematic and Molecular Basis of the Antibacterial Action of Quinoxaline 1,4-Di-N-Oxides against Escherichia coli
Source: PLoS One. 2015 Aug 21;10(8):e0136450. doi: 10.1371/journal.pone.0136450 (PMC4546592; doi:10.1371/journal.pone.0136450)
Supplement: S1 Text — (DOC) [file pone.0136450.s010.doc]

**S1 Text. Supplementary materials and methods**

**Identification of CYA metabolites in *E. coli***

*E. coli* CVCC2943 was treated with 4 µg/ml CYA for 0.5 h under aerobic or anaerobic (85% N_2_, 10% H_2_, and 5% CO_2_) conditions respectively, and the treated bacteria were added with 1 ml methanol and ultrasonicated for 2 min. After centrifugation, the supernatant was added with 1 ml of 15% trichloroacetic acid, followed by centrifugation at 10,000 *g* for 10 min. Then, the supernatant was dried under a stream of nitrogen at 50°C, followed by addition of 500 μl 20% (vol/vol) methanol and mixed thoroughly. The solution was filtered through a 0.22 μm nylon membrane, and aliquots were subjected to the high performance liquid chromatography-ion trap-time of flight mass spectrometer (HPLC/ESI-IT-TOF MS) (Shimadzu, Kyoto, Japan) for analysis according to (Xu et al., Rapid Communications in Mass Spectrometry 2011; 25: 2333-2344).

**Detection of ·OH radicals**

The QdNO-treated bacteria were resuspended in 500 µl of PBS containing 10 μM HPF, and incubated in the dark at 37 °C for 15 min. The QdNO-treated bacteria (5 μg/ml carbenicillin as positive control) were resuspended in 350 µl PBS for fluorescence measurement using CyAn ADP flow cytometer (Beckman Coulter, Brea, CA, USA) with excitation and emission wavelengths of 490 and 530 nm respectively. At least 10,000 cells were counted for each sample.
